# ^17^O solid-state NMR spectroscopy of A_2_B_2_O_7_ oxides: quantitative isotopic enrichment and spectral acquisition?†
†Electronic supplementary information (ESI) available. See DOI: 10.1039/c8ra00596f


**DOI:** 10.1039/c8ra00596f

**Published:** 2018-02-14

**Authors:** Arantxa Fernandes, Robert F. Moran, Scott Sneddon, Daniel M. Dawson, David McKay, Giulia P. M. Bignami, Frédéric Blanc, Karl R. Whittle, Sharon E. Ashbrook

**Affiliations:** a School of Chemistry, EaStCHEM and Centre of Magnetic Resonance, University of St Andrews, St Andrews KY16 9ST, UK. Email: sema@st-andrews.ac.uk; b Department of Chemistry, Stephenson Institute for Renewable Energy, University of Liverpool, Crown Street, Liverpool, L69 7ZD, UK; c School of Engineering, University of Liverpool, Brownlow Hill, Liverpool, L69 3GH, UK

## Abstract

The potential of ^17^O NMR spectroscopy for the investigation of A_2_B_2_O_7_ ceramic oxides important in the encapsulation of radioactive waste is demonstrated, with post-synthetic enrichment by exchange with ^17^O_2_ gas. For Y_2_Sn_2_O_7_, Y_2_Ti_2_O_7_ and La_2_Sn_2_O_7_ pyrochlores, enrichment of the two distinct O species is clearly non quantitative at lower temperatures (∼700 °C and below) and at shorter times, despite these being used in prior work, with preferential enrichment of OA_2_B_2_ favoured over that of OA_4_. At higher temperatures, the ^17^O NMR spectra suggest that quantitative enrichment has been achieved, but the integrated signal intensities do not reflect the crystallographic 1 : 6 (O1 : O2) ratio until corrected for differences in *T*_1_ relaxation rates and, more importantly, the contribution of the satellite transitions. ^17^O NMR spectra of Y_2_Zr_2_O_7_ and Y_2_Hf_2_O_7_ defect fluorites showed little difference with any variation in enrichment temperature or time, although an increase in the absolute level of enrichment (up to ∼7.5%) was observed at higher temperature. DFT calculations show that the six distinct resonances observed cannot be assigned unambiguously, as each has contributions from more than one of the five possible next nearest neighbour environments. For La_2_Ti_2_O_7_, which adopts a layered perovskite-like structure, little difference in the spectral intensities is observed with enrichment time or temperature, although the highest absolute levels of enrichment (∼13%) were obtained at higher temperature. This work demonstrates that ^17^O NMR has the potential to be a powerful probe of local structure and disorder in oxides, but that considerable care must be taken both in choosing the conditions for ^17^O enrichment and the experimental acquisition parameters if the necessary quantitative measurements are to be obtained for more complex systems.

## Abstract 2

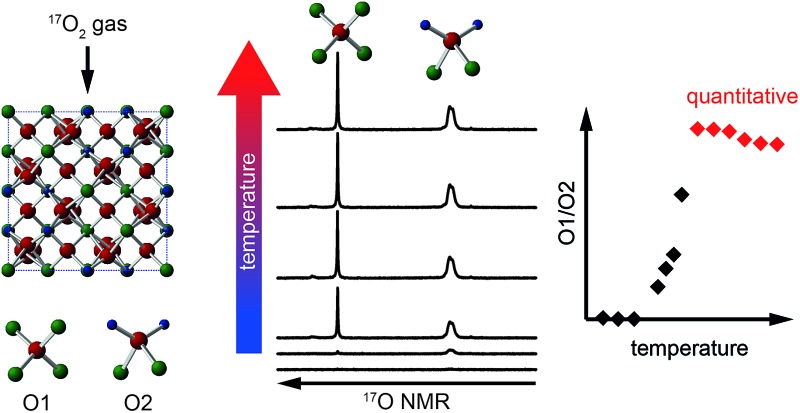
Investigation of the conditions required for quantitative isotopic enrichment and the acquisition of quantitative ^17^O NMR spectra of ceramic oxides.

## Introduction

The fundamental importance of oxide-based systems in technology, the energy arena, geochemistry and catalysis, and the presence of oxygen in a number of biomaterials, should have resulted in oxygen nuclear magnetic resonance (NMR) spectroscopy emerging as a vital tool for the characterisation of solid materials. As NMR provides an element-specific and atomic-scale measurement of the local environment, it is potentially a powerful probe of the local structure, disorder and dynamics in solids.^1^–^4^ However, despite the widespread presence of oxygen in inorganic solids, oxygen NMR studies are much less common in comparison to more easily studied nuclei, owing primarily to the low natural abundance of the only NMR-active isotope, ^17^O (0.037%).^5^–^7^ Furthermore, the presence of anisotropic quadrupolar broadening (*I* = 5/2) often necessitates the use of more complex (and more time-consuming) experiments to obtain high-resolution spectra.^8^,^9^

In many cases, isotopic enrichment in ^17^O (either of the starting materials prior to reaction, or of the final product itself) is required to enable NMR spectra to be acquired on a reasonable timescale, particularly for multidimensional experiments.^2^,^5^–^7^ However, this can be extremely costly, and can necessitate the alteration of synthetic approaches to develop cost- and atom-efficient protocols. These often include the reduction or removal of water from a synthesis (*e.g.*, the use of dry gel conversion reactions,^10^,^11^ steaming procedures,^11^,^12^ ionothermal synthesis^13^ or mechanochemistry^14^). However, for many oxides, enrichment is typically carried out post synthesis, by heating in ^17^O_2_ gas,^2^,^5^,^7^ although the literature often provides little information about why the times and temperatures used were chosen, or on the eventual enrichment level achieved (although this can be very difficult to determine accurately). In most cases, the principal objective is to obtain a sufficiently high level of enrichment that the desired NMR experiments become feasible, but a second aim must be to understand whether the enrichment achieved is quantitative, *i.e.*, whether all types of O species are equally and uniformly enriched. Although selectivity in an enrichment process may provide useful information about mechanism or reactivity, in other cases, *e.g.*, when trying to understand disorder, quantitative enrichment is required to ensure all regions or all phases of a sample are equally represented. The inherently quantitative nature of NMR spectroscopy is an invaluable property for its use in structure determination, particularly for materials that lack the long-range order required by other techniques.^4^ However, for quadrupolar nuclei, the acquisition of quantitative spectra poses additional challenges, with even simple magic-angle spinning (MAS) spectra, showing non-uniform excitation of species with different magnitudes (*C*_Q_) of the quadrupolar interaction, owing to a variation in nutation frequency.^1^,^2^,^8^,^9^ Although corrections can often be made for such excitation inefficiencies by comparison to simulation, this issue does make it difficult to determine easily whether any synthetic enrichment process is quantitative unless more detailed studies are performed.

Here, we consider the isotopic enrichment of ceramic oxides with general formula A_2_B_2_O_7_, where A = Y or La and B = Sn, Hf, Ti or Zr, which find applications in a wide range of areas such as electronics, in the energy materials and for the encapsulation of radioactive waste.^15^–^19^ The lack of ^1^H in these materials and their bulk (rather than nanoparticulate) nature mean that popular sensitivity enhancement approaches in NMR spectroscopy, such as the use of cross polarisation (CP)^20^ or dynamic nuclear polarization (DNP),^21^,^22^ are unfeasible, often leaving isotopic enrichment as the only practical alternative. The materials considered here adopt three distinct structure types depending upon the radii of the A and B cations. When *r*_A_/*r*_B_ is between 1.46 and 1.78 a pyrochlore phase is typically formed.^23^,^24^ A decrease in *r*_A_/*r*_B_ results in the formation of a defect fluorite phase, and an increase in this ratio gives a layered material similar to perovskite.^23^,^24^ The chemical and physical properties of these oxides can be altered by cation substitution, although the behavior observed will depend upon the cation distribution (both relative to each other and to any oxygen vacancies/positions). In some cases, substitution is predicted to result in a more significant change in structure, leading to questions on the limits of any solid solution behaviour, or on the presence, amount and composition of materials in multi-phase mixtures. The sensitivity of NMR spectroscopy to the local, atomic-scale environment, without the need for any long-range order ensures it is an ideal tool for studying the structure of disordered materials, with the acquisition of quantitative spectra playing a vital role.^2^,^4^ NMR spectroscopy has been applied to the study of A_2_B_2_O_7_ ceramics on many occasions,^25^–^35^ with early work investigating Ln^3+^ cation substitution in (Y,Ln)_2_M_2_O_7_ (where M = Sn or Ti) pyrochlores.^25^,^26^ More recent work combines experiment with first-principles calculations to examine the cation distribution in Y_2_(Sn,Ti)_2_O_7_ and La_2_(Ti,Sn)_2_O_7_ (ref. 29–33) and to determine the number, proportion and composition of phases present in Y_2_(Sn,Zr)_2_O_7_ (ref. 34) and La_2_(Ti,Sn)_2_O_7_,^35^ where structural changes are predicted from pyrochlore to defect fluorite and layered perovskite-like materials, respectively. NMR spectra of ^89^Y and ^119^Sn (both *I* = 1/2) provide only an indirect measure of the cation distribution in such materials, *i.e.*, *via* changes in the next nearest neighbor (NNN) species, and these nuclides are not present equally in every material, making it challenging to determine accurately the proportion of each phase present in multi-component systems. For some materials, no spin *I* = 1/2 nuclei are readily available for study, and the quadrupolar nuclei present have very large quadrupole moments (*e.g.*, ^91^Zr, ^139^La, ^47/49^Ti, *etc.*), necessitating the use of wideline experiments, limiting the spectral resolution and, consequently, the information available.^8^,^9^ ^17^O NMR should provide a valuable additional characterization tool for the study of these ceramics, as it is present (in equal amounts) in every A_2_B_2_O_7_ phase, and is directly coordinated to the cations of interest, providing a much more direct measure of their distribution. However, in order to achieve this, uniform enrichment and the acquisition of quantitative spectra (or at least a knowledge of how to correct the relevant relative intensities) are required.

In this work, we provide a detailed investigation of ^17^O isotopic enrichment of pyrochlore, defect fluorite and layered perovskite-like A_2_B_2_O_7_ ceramics, exploring whether post-synthetic enrichment with ^17^O_2_ gas is uniform for the different O species (and under what conditions this can be achieved), and whether the acquisition of quantitative spectra is possible. We compare the conditions required for different end members (*i.e.*, stoichiometric A_2_B_2_O_7_), as a first step to the longer-term, and more challenging, aims of studying cation disorder and/or multiphase mixtures. Using first-principles calculations the observed O resonances are assigned either to crystallographically distinct species, or to a particular type of chemical environment. This work represents a vital pre-requisite for the study of more complex ceramics demonstrating the need for considerable care both when enriching these materials and when acquiring or interpreting spectra, and the need to state the conditions used or chosen explicitly. However, the results obtained indicate that detailed ^17^O NMR investigation has the potential to provide a wealth of information on structure, composition and disorder in these important ceramic oxides.

## Experimental details

### Synthesis

Y_2_Sn_2_O_7_, Y_2_Ti_2_O_7_, La_2_Sn_2_O_7_, Y_2_Zr_2_O_7_, Y_2_Hf_2_O_7_ and La_2_Ti_2_O_7_ were prepared using conventional solid-state synthesis. La_2_O_3_ (Sigma-Aldrich 99.9%), TiO_2_ (Sigma-Aldrich 99%), SnO_2_ (Sigma-Aldrich 99.9%), Y_2_O_3_ (Sigma-Aldrich 99%), ZrO_2_ (Sigma-Aldrich 99%) or HfO_2_ (Sigma-Aldrich 99%) were pre-fired in a muffle furnace at 800 °C for 12 h to burn off any residues of impurities, hydroxides, CO_2_ and H_2_O. Stoichiometric amounts of the powders were then activated by ball milling at 600 rpm for 1 h in acetone with zirconia media, dried and (uniaxially) pressed into pellets. The pellets were then heated at 1400 °C for 48 h (with intermediate regrinding and repressing), with a ramp rate of 10 °C min^–1^. After cooling, the samples were ground for both X-ray diffraction (XRD) and MAS NMR.

### X-ray diffraction and microscopy

Samples were characterised by powder XRD using a PANalytical Empyrean, with weighted CuK_α1_ (*λ* = 1.540598 Å) radiation over an angular range of 10° to 100° in 2*θ* with a step size of 0.02° and a step duration of 0.4 s. Powder XRD patterns can be found in the ESI.† SEM measurements were carried out using a JSM-5600 conventional scanning electron microscope with a tungsten filament electron source, equipped with a secondary electron detector for topographic contrast imaging and an Oxford Inca EDX system for compositional analysis. A working distance of 20 mm, a spot size of 40 and a voltage of 25 kV in the W filament were used. Samples were placed on sample holders and gold coated with a Quorum Q150R ES instrument to provide a conductive surface. Images are given in the ESI (Fig. S1.7†).

### Isotopic enrichment

Samples were enriched by condensing ∼0.03 L of 70% ^17^O_2_(g) (Cortecnet) into a pre-evacuated quartz vial containing ∼0.4 g of oxide, before heating in a tube furnace at temperatures between 150–950 °C for between 6 and 96 h. A ramp rate of 5 °C min^–1^ was used for heating and cooling. After enrichment, the powder XRD and ^89^Y NMR measurements (shown in the ESI, S2†) were re-recorded to ensure there were no significant changes in the samples.

### Mass spectrometry

Samples were prepared by embedding the powdered materials in indium (99.9995% purity) mounts using a hydraulic press (*ca.* 5 ton) then covered with a gold coat *ca.* 30 nm thick. The oxygen isotope data were acquired at the University of Edinburgh using a Cameca ims 1270. A ^133^Cs^+^ focussed primary ion beam of ∼4 nA was rastered over an area of 25 μm^2^. Secondary ions were extracted at 10 kV and ^16^O, ^17^O and ^18^O were monitored at a mass resolution of ∼6000 using either a Faraday cup or an electron multiplier depending on the count rates of the isotope. Background, relative detector yield and dead-time corrections were applied to the count-rates recorded. Each analysis involved a pre-sputtering time of 60 s, followed by automatic centring of the secondary ion beam into the field aperture (3000 μm) and entrance slits (30 μm). Isotopic data was acquired in two blocks of ten cycles, amounting to a total count time of 80 s per isotope. Results were obtained with standard error of the mean in the range 0.2–0.8% depending on the uniformity of the sample surface. The instrument calibration and alignment was checked by measuring the isotopic ratio of a natural abundance mineral (ilmenite) standard. Average enrichment levels of ∼5%, ∼7.5% and ∼13% were obtained for samples of Y_2_Sn_2_O_7_ (enriched at 900 °C for 12 h), Y_2_Hf_2_O_7_ (enriched at 900 °C for 24 h) and La_2_Ti_2_O_7_ (enriched at 800 °C for 12 h), respectively. More detail is given in the ESI (Tables S1.1–S1.3†).

### NMR spectroscopy

^17^O MAS NMR spectra were acquired using Bruker Avance III spectrometers equipped with 9.4 T, 14.1 T or 20.0 T wide-bore magnets, operating at ^17^O Larmor frequencies of 54.243 MHz, 81.356 MHz and 115.248 MHz, respectively. At 20.0 T and 14.1 T, powdered samples were packed into a 3.2 mm ZrO_2_ rotor and rotated at MAS rates of 20–21 kHz. At 9.4 T, samples were packed into a 4.0 mm ZrO_2_ rotor and rotated at an MAS rate of 13.5 kHz. Spectra were acquired at room temperature using a radiofrequency field strength, *ν*_1_ of ∼70 kHz, and a recycle interval of 5 s (pyrochlore, layered perovskite) or 1 s (defect fluorite). Unless otherwise stated, experiments were carried out using a short flip angle (π/14) of 0.5 μs. Chemical shifts are shown (in ppm) relative to H_2_O recorded at 298 K. Fitting of the spectral lineshapes was carried out using the SOLA program (available with the Topspin software). *T*_1_ measurements were performed using a saturation recovery experiment (also with a short flip angle pulse), with a typical saturation train of 100 pulses separated by intervals of 10 ms. Experimental intensities were compared to those in spectra simulated using the density matrix simulation program SIMPSON.^36^ In these simulations, spectra were simulated at 14.1 T under the experimental conditions described above using detection operators of I_1x_ (*i.e.*, all ^17^O single-quantum coherences from the central transition (CT) and satellite transitions (ST)) or I_1c_ (*i.e.*, only ^17^O CT coherences). Simulations were carried out using 100 × 320 angles. ^17^O MQMAS spectra were obtained at *B*_0_ = 14.1 T and 20.0 T using a z-filtered pulse sequence^37^,^38^ with a CT-selective (*ν*_1_ of ∼3 kHz) 90° pulse. Spectra are referenced in the indirect dimension according to the convention described in ref. 39.

### Calculations

Periodic density functional theory (DFT) calculations were carried out using CASTEP (version 8.0).^40^ Calculations were performed using the PBE^41^ exchange correlation functional and core-valence interactions were described by ultrasoft pseudopotentials,^42^ accounting for scalar relativistic effects using ZORA. A planewave energy cutoff of 50 Ry was used. The first Brillouin zone was sampled through a Monkhorst–Pack grid^43^ with a k-point spacing of 0.05 2π Å^–1^. Initial structural models were obtained from the literature, and were optimised prior to the calculation of NMR parameters, with all atomic coordinates and cell parameters allowed to vary. NMR parameters were then computed employing the gauge-including projector augmented wave (GIPAW)^44^ approach to reconstruct the all-electron wavefunction in the presence of a magnetic field. Calculations were performed on a cluster at the University of St Andrews, consisting of 300 12-core Intel Westmere nodes, connected with QDR Infiniband. Diagonalisation of the symmetric part of the absolute shielding tensor (***σ***), yields the three principal components, *σ*_11_, *σ*_22_ and *σ*_33_, from which the isotropic shielding can be determined by *σ*_iso_ = (*σ*_11_ + *σ*_22_ + *σ*_33_)/3. Typically, the computed isotropic shift, *δ*calciso, is determined from this to allow comparison to experimentally measured shifts. The procedures used in this work are described in detail in the ESI (S3†). Diagonalisation of the electric field gradient tensor, ***V***, gives principal components *V*_*XX*_, *V*_*YY*_ and *V*_*ZZ*_ (ordered such that |*V*_*ZZ*_| ≥ |*V*_*YY*_| ≥ |*V*_*XX*_|). From these the magnitude of the quadrupolar interaction, *C*_Q_ = *eQV*_*ZZ*_/*h*, where *Q* is the nuclear quadrupole moment (for which a value of 25.58 mb was used for ^17^O) and the asymmetry parameter, *η*_Q_ = (*V*_*XX*_ – *V*_*YY*_)/*V*_*ZZ*_ can be determined. The quadrupolar product, *P*_Q_ = *C*_Q_(1 + *η*_Q_^2^/3)^1/2^.

## Results and discussion

### Pyrochlores

**Fig. 1:**
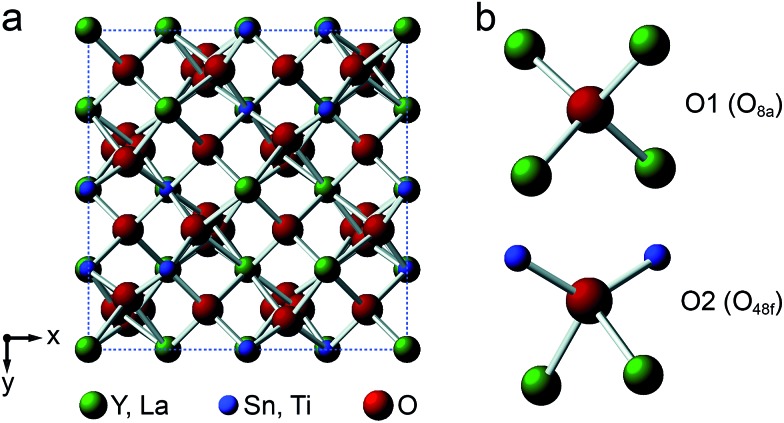
(a) Structure of a typical A_2_B_2_O_7_ pyrochlore. Red spheres denote O, green spheres, the eight-coordinate A site (occupied by Y or La), and blue spheres, the six-coordinate B site (occupied by Ti or Sn). (b) Expansions showing the coordination environments of the O1 (8a) and O2 (48f) sites.

Y_2_Sn_2_O_7_, Y_2_Ti_2_O_7_ and La_2_Sn_2_O_7_ all adopt the pyrochlore structure (*Fd*3*m* symmetry), derived from that of fluorite (AO_2_) by the ordered removal of 1/8 of the O atoms.^23^,^24^ As shown in Fig. 1a, this results in two types of cation species: an eight-coordinate A site and a six-coordinate B site. As shown in Fig. 1b, there are three distinct O positions within the unit cell, the 48f (or O2) site, where oxygen is coordinated by two A and two B cations, the 8a (or O1) site, where all four coordinating atoms are A cations, and the (now vacant) 8b site (note that for some crystal structures, the definition of O1 and O2 is reversed, *i.e.*, O1 is 48f and O2 is 8a, but we have chosen to follow the crystallographic notation used in ref. 45).

**Fig. 2:**
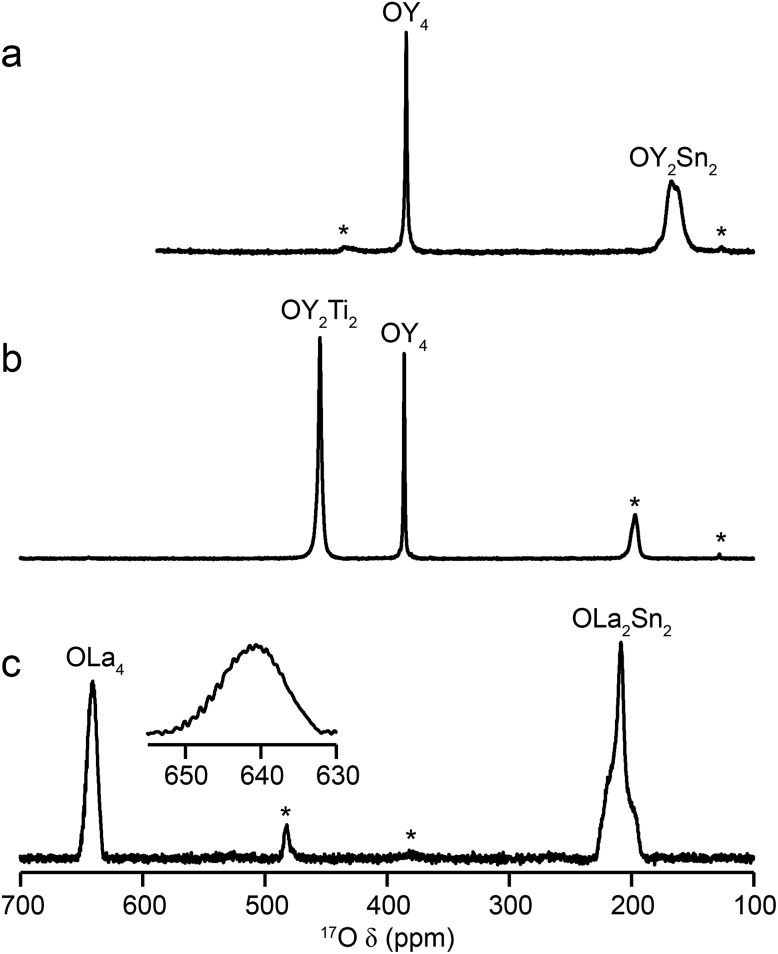
^17^O (14.1 T, 21 kHz) MAS NMR spectra of (a) Y_2_Sn_2_O_7_ (b) Y_2_Ti_2_O_7_ and (c) La_2_Sn_2_O_7_ pyrochlores (enriched at 700 °C for 12 h), with the O coordination environments indicated. Spinning sidebands are shown by *. In (c), the lineshape of the OLa_4_ resonance is shown expanded.

Fig. 2 shows ^17^O MAS NMR spectra of the three pyrochlore materials enriched at 700 °C for 12 h. Two signals are observed in all cases, one typically much sharper than the second, reflecting a smaller *C*_Q_ value. NMR parameters extracted from the spectra are given in Table 1. The symmetry of the coordination environments in Fig. 1b suggests that the resonance with very low *C*_Q_ results from O1, as this has OA_4_ coordination, while O2 has a less symmetrical OA_2_B_2_ local environment. This is supported by first-principles DFT calculations, which predict that the O1 *C*_Q_ should be 0 for all three pyrochlores (reflecting the formal 43*m* point symmetry at the 8a site), but gives non-zero *C*_Q_ values for the O2 species, as shown in Table 1. The calculated *C*_Q_ and *η*_Q_ values for O2 are in good agreement with the experimental measurements (determined for Y_2_Sn_2_O_7_ and La_2_Sn_2_O_7_ by fitting of the second-order quadrupolar broadened lineshapes, and for Y_2_Ti_2_O_7_ by fitting the sideband manifold in spectra acquired at a slow MAS rate). As discussed in ref. 5 and 27, the magnitude of the O2 *C*_Q_ value reflects the covalent character of the M–O bonds rather than any distortion of the OA_2_B_2_ polyhedra (the latter resulting from the deviation of the 48f oxygen position (*x*, 1/8, 1/8) from that in ideal fluorite (3/8, 1/8, 1/8)). As shown in Table 1, the O2 *C*_Q_ values for the two stannate pyrochlores (with more covalent O–Sn) bonds are considerably higher than that in Y_2_Ti_2_O_7_. Although, in principle, a *C*_Q_ of 0 is expected for all O1 species, ST spinning sidebands are observed in the experimental spectra, indicating a (small) non-zero electric field gradient at O1, most likely arising from defects and/or surface effects within the material. As shown in the ESI (S4†), the coupling constant can be estimated by fitting the sideband manifold in spectra acquired at slow (*i.e.*, 3.25 kHz) MAS rates. The spectra in Fig. 2 are in good agreement with those in previous literature for Y_2_Sn_2_O_7_ and Y_2_Ti_2_O_7_ (note the reversal of the definition of O1 and O2 used in ref. 27), although exact *C*_Q_ values for the OY_4_ environments were not provided.

**Table 1:** Experimental and calculated ^17^O NMR parameters for Y_2_Sn_2_O_7_, Y_2_Ti_2_O_7_ and La_2_Sn_2_O_7_ pyrochlores

Pyrochlore	Site	Experimental			Calculated			
		*δ*_iso_ (ppm)	*C*_Q_^*b*^/MHz	*η*_Q_	*σ*_iso_ (ppm)	*δ*calciso ^*a*^ (ppm)	*C*calcQ ^*b*^/MHz	*η*calcQ
Y_2_Sn_2_O_7_	O1	384.0	∼0.02	0.14	–184.02	394.5	0.0	—
	O2	172.5	3.2 (1)	0.36	31.87	200.1	3.52	0.35
Y_2_Ti_2_O_7_	O1	386.1	∼0.02	0.13	–171.3	383.0	0.0	—
	O2	454.6	0.7 (1)	0.50	–256.87	460.0	–0.64	0.76
La_2_Sn_2_O_7_	O1	641.5	∼0.02	0.20	–320.89	517.7	0.9	—
	O2	222.0	3.3 (1)	0.90	–6.84	235.0	3.8	0.99

^*a*^See ESI (S3) for information on referencing of the calculations.

^*b*^Note that the experiment can only measure the absolute value of *C*_Q_, while the sign can be determined from calculation.

For La_2_Sn_2_O_7_, expansion of the signal from the O1 site (shown inset in Fig. 2c) reveals a very complex lineshape. This oxygen should exhibit *J* couplings to four equivalent ^139^La (*I* = 7/2) species (ideally producing a 29 (*i.e.*, 2*NI* + 1) line multiplet). However, a symmetrical multiplet is not observed, owing to the large ^139^La quadrupolar coupling constant (predicted by DFT calculations to be ∼76 MHz and measured experimentally using wideline CPMG^46^,^47^ experiments as ∼79 MHz – see the ESI (S5†) for more detail). This (along with the close proximity of the two spins) results in a quadrupolar–dipolar cross term (sometimes termed “residual dipolar coupling”) that, as a second-order interaction, survives under MAS.^48^,^49^ This interaction both alters the splittings of the multiplet (which are no longer all equal to *J*) and causes each component of the multiplet to exhibit a powder-pattern lineshape. The exact pattern and spacing observed depends upon the magnitude of the dipolar, scalar and quadrupolar couplings, the MAS rate, the anisotropic components of the interactions involved and the relative orientation of the interaction tensors. Although the lineshape also varies with *B*_0_ field strength (as expected for a quadrupolar–dipolar cross term), as shown in the ESI (S5†), resolution is not sufficient to fit the lineshape or experimentally determine the couplings involved in this case.

**Fig. 3:**
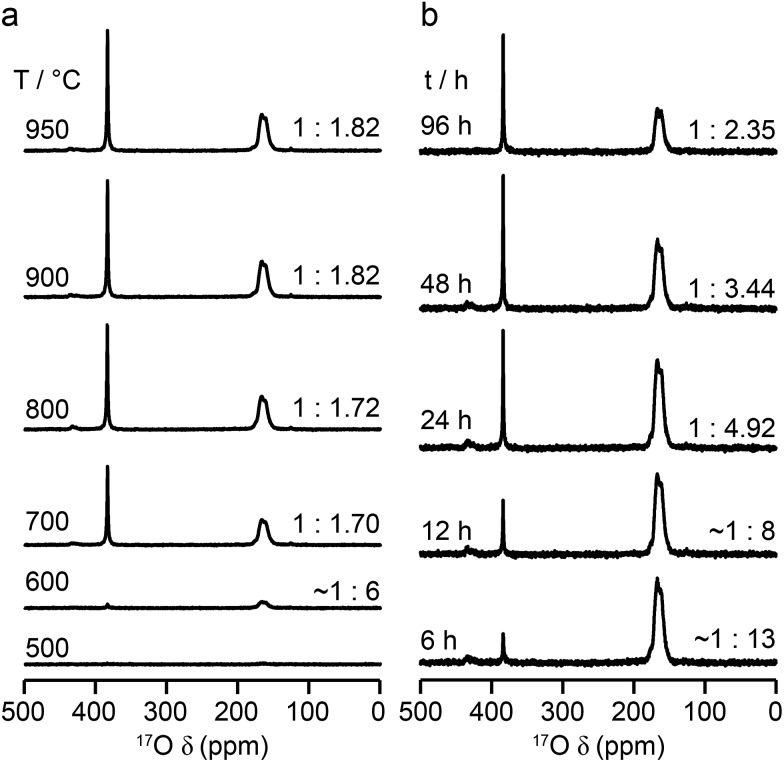
^17^O (14 T, 21 kHz) MAS NMR spectra of Y_2_Sn_2_O_7_ enriched (a) for 12 h at the temperatures shown and (b) at 600 °C for the durations shown. Spectra were acquired using the same mass of sample and by averaging the same number of transients (note that the two spectra enriched at 600 °C are from different samples and the error on this measurement is greater owing to the low level of enrichment).

**Fig. 4:**
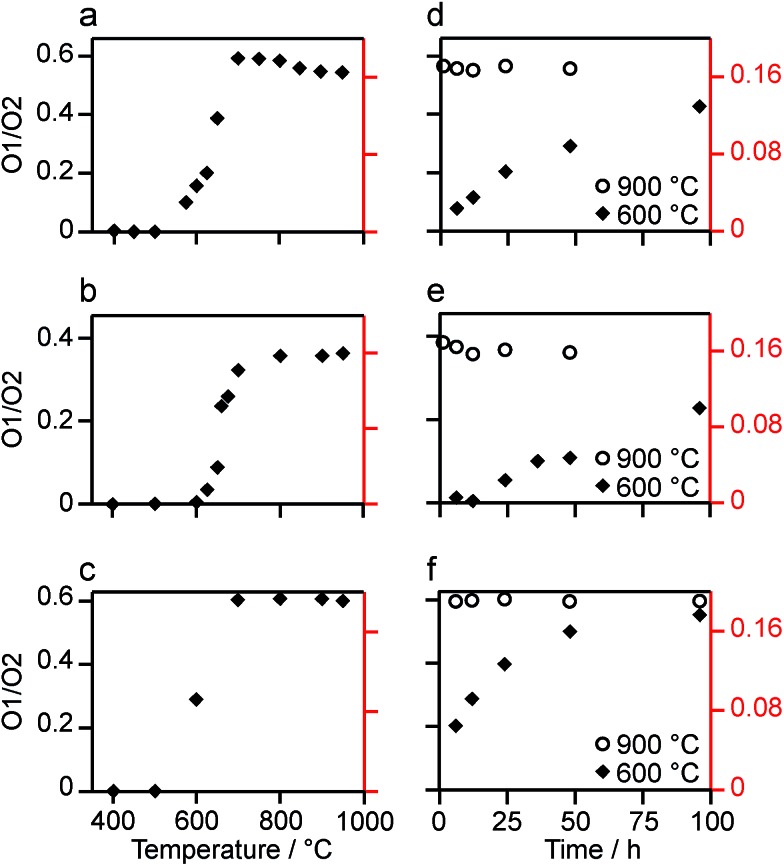
Plots showing the (integrated) intensity ratio, O1/O2, extracted from ^17^O MAS NMR spectra of (a, d) Y_2_Sn_2_O_7_, (b, e) Y_2_Ti_2_O_7_ and (c, f) La_2_Sn_2_O_7_, enriched (a–c) for 12 h at the temperatures shown and (d–f) at 600 °C (diamonds) and 900 °C (circles) for the durations shown. Axes in red show the O1/O2 ratio corrected for the effects of relaxation and the contribution of the ST (see text).

The relative intensities of the resonances for the O1 and O2 species in a pyrochlore would be expected to be 1 : 6 (Wykoff positions 8a and 48f, respectively).^23^,^24^ However, the spectra in Fig. 2 for Y_2_Sn_2_O_7_, Y_2_Ti_2_O_7_ and La_2_Sn_2_O_7_ have ratios of 1 : 1.7, 1 : 3.1 and 1 : 1.7, respectively, indicating that enrichment is non uniform at the temperature used (700 °C), or that the spectral acquisition was not quantitative. To investigate this further, ^17^O enrichment was carried out at a variety of temperatures between 400 °C and 950 °C (for 12 h in each case). The resulting ^17^O MAS NMR spectra for Y_2_Sn_2_O_7_ are shown in Fig. 3a (no spectrum was obtained for the sample enriched at 400 °C). At low temperature, little total ^17^O signal is observed, confirming the low level of exchange. The overall signal intensity increases as the enrichment temperature is raised, and higher levels of ^17^O are incorporated (note that the same mass of sample was used in all cases). More importantly, changes are also seen in the O1 : O2 ratio with temperature, *i.e.*, in the relative levels of enrichment (which varies from ∼1 : 6 at 600 °C to 1 : 1.82 at 950 °C), as shown in Fig. 4a (plotted numerically as O1/O2). For the two yttrium-containing pyrochlores, the ratios observed here are also consistent with those from previous work for material enriched under the same conditions. Although an O1 : O2 ratio of ∼1 : 6 (0.1667) is observed at lower temperatures, it is clear that a constant relative enrichment of the two sites is only achieved at much higher temperatures. This suggests that not only is the rate (and total level) of enrichment of the two sites different, but that perhaps a quantitative spectrum has not been acquired (see later discussion). A similar result is obtained for Y_2_Ti_2_O_7_ and La_2_Sn_2_O_7_ (see ESI (S6†) for spectra and Fig. 4b and c for changes in the O1/O2 ratio), with the total level of ^17^O enrichment and the O1/O2 ratio increasing as the temperature of enrichment increases. A constant O1/O2 ratio is observed at different temperatures for the three pyrochlores, but is achieved above ∼800 °C in each case. It is worth noting at this point that the O1/O2 ratio at high temperature is similar for Y_2_Sn_2_O_7_ and La_2_Sn_2_O_7_ but much higher for Y_2_Ti_2_O_7_.

Fig. 3b reveals that the relative enrichment level of the two O species in Y_2_Sn_2_O_7_ (heated at 600 °C) also varies with the heating duration, with much more signal from O2 observed at shorter times and relatively less as the time increases. In contrast, and as shown in Fig. 4d, when enrichment is carried out at ∼900 °C the O1/O2 ratio is constant no matter what the heating duration (although the absolute level of enrichment does still increase). Similar results are found for Y_2_Ti_2_O_7_ and La_2_Sn_2_O_7_ (see ESI (S6†) for spectra and Fig. 4e and f for changes in the O1/O2 ratio). For Y_2_Sn_2_O_7_, for the O1/O2 ratio to reach that seen at ∼900 °C, enrichment times of over ∼120 h would be required at ∼600 °C. Although this would be a much longer procedure, there may be advantages to enriching at lower temperatures if this minimises or prevents structural changes (*i.e.*, phase transitions or reordering in the case of disordered materials) that may occur at higher temperatures.

Although the spectra of pyrochlore samples enriched at higher temperatures (or for longer times) exhibit a constant O1/O2 ratio, this does not equal the 1 : 6 expected from the structure. This suggests that either there is a fundamental difference in the maximum level of enrichment possible for the two sites or, more likely, that the approach used to acquire the spectra is in itself non quantitative. Given the quadrupolar nature of ^17^O, one possible explanation is that the O1 and O2 exhibit different CT nutation rates, leading to a non-uniform excitation of the two.^1^,^2^,^8^,^9^ However, nutation experiments and simulations using SIMPSON (see ESI (S7†)) reveal that the short flip angles used in the spectral acquisition (formally π/14) are sufficiently small that no differences in excitation efficiency are apparent. It should be noted that for longer pulse durations (typically >0.8 μs or >π/9) differences in nutation would have to be taken into account. A second possibility for a non-uniform excitation is differences in the *T*_1_ relaxation rates of the two O species. *T*_1_ relaxation rates were measured for each O site in the three pyrochlore samples using saturation recovery experiments, and values are given in Table 2. The values suggest that fully quantitative spectra would only be obtained with very long recycle intervals. Interestingly, *T*_1_ relaxation is slowest for Y_2_Sn_2_O_7_ and fastest for La_2_Sn_2_O_7_, and for the Y-based pyrochlores *T*_1_ relaxation is slowest for the O1 (OY_4_) environments. In contrast, for La_2_Sn_2_O_7_, relaxation is faster for O1 (OLa_4_) and slower for O2 (OLa_2_Sn_2_), although both *T*_1_ time constants are much smaller (*i.e.*, more rapid relaxation) for this phase. Given the structural similarities, these differences most likely result from the close proximity of quadrupolar nuclei (^47/49^Ti for O2 in Y_2_Ti_2_O_7_ and ^139^La in La_2_Sn_2_O_7_), with more rapid relaxation. Although it is clear that there are some differences in relaxation (and, therefore, in signal intensity) at the recycle intervals used in the experiments, these can be estimated from the saturation recovery results, and accounted for when calculating O1/O2, with increases of 15, 33 and 20% for Y_2_Sn_2_O_7_, Y_2_Ti_2_O_7_ and La_2_Sn_2_O_7_, respectively.

**Table 2:** ^17^O *T*_1_ relaxation time constants (measured using saturation recovery experiments with a short flip angle pulse) for the two O species in Y_2_Sn_2_O_7_, Y_2_Ti_2_O_7_ and La_2_Sn_2_O_7_ pyrochlores

Pyrochlore	Site	*T*_1_/s
Y_2_Sn_2_O_7_	O1	367 (5)
	O2	295 (5)
Y_2_Ti_2_O_7_	O1	230 (5)
	O2	71 (3)
La_2_Sn_2_O_7_	O1	12 (2)
	O2	20 (1)

Even when relaxation differences are accounted for the O1/O2 ratio does not reflect the expected 1 : 6 expected from the structural model. However, owing to the very small (*i.e.*, almost 0) *C*_Q_ observed for O1 in all three pyrochlores, it is likely that some of the signal intensity observed for this species results not from the CT but from the centreband of the STs.^1^,^2^,^8^,^9^ Note that as *C*_Q_ increases, a (second-order) isotropic quadrupolar shift of both CT and ST is observed (hence, fitting of the spectrum is usually required to determine the isotropic chemical shift, *δ*_iso_, as the resonance is slightly shifted from this position). For species experiencing a large quadrupolar interaction, these isotropic shifts separate the CT and the ST centreband in the spectrum, and the integrated intensity of the CT resonance can be unambiguously determined. It is possible to simulate the contribution of CT and ST to the spectrum (at the rf field strengths, pulse durations and MAS rates used) for each of the O sites in each pyrochlore using a density matrix based approach, as implemented in SIMPSON^36^ (see Experimental section). For Y_2_Sn_2_O_7_ and La_2_Sn_2_O_7_, the STs contribute only to the O1 signal (which has a negligible *C*_Q_) and not to the O2 signal, while for Y_2_Ti_2_O_7_, the contribution of the ST to the O2 CT signal must also be taken into account. When this additional correction is implemented the CT O1/O2 ratio can be seen to be close to the 1 : 6 ratio expected from the structure, as shown by the alternative (red) axes in Fig. 4.

Although Fig. 4 shows that uniform enrichment of O1 and O2 species can be achieved, it is clear that the rate of enrichment is different for the two, with preferential enrichment of O2 at lower temperatures and/or shorter times. It should also be noted that the rate at which the O1/O2 ratio approaches its constant value is more rapid for the two stannate pyrochlores. Differences in the rate of ^17^O enrichment have been observed previously for the reaction of the zeolite stilbite with water vapour.^50^ In this system, the diffusion of water vapour is so rapid that any differences in exchange rates could be related directly to the site exchange kinetics. It was observed that Si–O–Si species exchanged relatively slowly, Si–O–Al sites more rapidly, and Al–O–Al sites underwent very rapid exchange. Further work showed a similar result for analcime,^51^ with Si–O–Al sites exchanging much more rapidly than Si–O–Si sites at lower temperature (400 °C), while at 500 °C the exchange rates were similar.

For the three pyrochlores studied here, the rate of ^17^O enrichment is greatest at lower temperatures or shorter times for the 48f/O2 oxygen, which is coordinated by two A and two B cations, and is slower for the 8a/O1 oxygen, which has an OA_4_ coordination. This could reflect the general difference in bond dissociation energies and ionicity,^52^ with Sn–O and Ti–O bonds having lower dissociation energies and a greater covalency, ensuring O2 sites are typically easier to exchange than O1. However, at lower temperatures it may well be that the isotopic exchange is also affected by the diffusion rate in these dense ceramics (as opposed to the porous zeolite materials described above). It is known that the mechanism of oxygen migration in pyrochlores is thought to be defect mediated, and proceeds *via* exchange of vacancies on neighbouring 48f oxygen sites (along the 100 and 110 directions).^53^–^55^ Recent computational work has demonstrated that for some pyrochlores, the nearby vacant 8b sites also plays a role in the ion migration, forming a so-called “split vacancy”.^53^–^55^ It should also be noted that the 8a oxygen sites are more isolated within the pyrochlore structure, both from each other and from the vacant 8b sites. Computational studies suggested that for La_2_Sn_2_O_7_ and Y_2_Ti_2_O_7_ the single vacancy configuration was more stable, whereas for Y_2_Sn_2_O_7_ the vacant 8b site played a greater role. The activation energies calculated for oxygen ion migration were similar for the two stannate pyrochlores (0.85 and 0.9 eV), but were higher for Y_2_Ti_2_O_7_ (1.27 eV). Although it is difficult to make any detailed conclusions or comparisons from the ^17^O spectra obtained here, given the practical challenges (*i.e.*, experimental uncertainties in the exact amount of gas used in each enrichment, small differences in the microstructure/particle size of the different ceramics, and the small differences in the absolute amounts of material used in the NMR experiments), it seems clear that the proposed mechanism of ion migration could also favour more rapid enrichment of O2. The differences observed between the pyrochlores, both in the rate at which a constant O1/O2 ratio is reached, and in the absolute, rather than relative, rates of enrichment (see ESI (S8†)), may well reflect both the exchange kinetics at each O site and the diffusion mechanism, making it difficult to explain, or indeed predict, the behaviour observed. The most important conclusion, however, is that, although the rate of exchange is different for O1 and O2 at low temperatures and shorter times, it is possible to obtain equally efficient enrichment of the two sites at higher temperature and/or longer heating times. Given the differences observed between these end-member pyrochlores it will, therefore, be vital for the ^17^O NMR study of disordered materials to enrich at higher temperatures.

### Defect fluorite

**Fig. 5:**
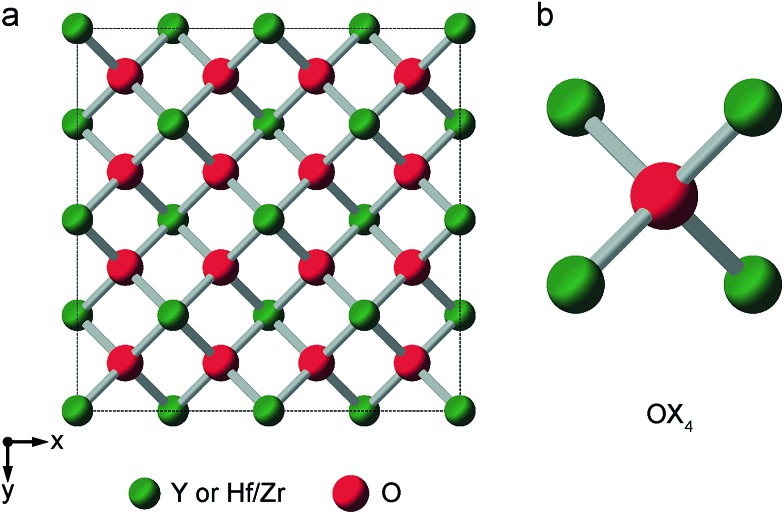
(a) Structure of a typical A_2_B_2_O_7_ defect fluorite material. Red spheres denote O sites (1/8 of which will be vacant) and green spheres the single cation site. (b) Expansion showing the oxygen coordination environment, where the cation sites can be occupied either by A or B cations.

When the *r*_A_/*r*_B_ ratio falls below 1.46, A_2_B_2_O_7_ systems typically form a defect fluorite phase (*Fm*3*m* symmetry), with disorder on both cation and anion lattices, as shown in Fig. 5.^23^,^24^ The two types of cation occupy the 4a position, and all (8c) oxygens are crystallographically identical, and coordinated by four cations. However, locally, five different oxygen environments are possible – OA_4_, OA_3_B, OA_2_B_2_, OAB_3_ and OB_4_. Y_2_Zr_2_O_7_ and Y_2_Hf_2_O_7_ adopt defect fluorite, rather than pyrochlore structures. Y_2_Zr_2_O_7_ has been previously studied using ^89^Y NMR spectroscopy,^28^,^34^ with three broad resonances (at ∼290, ∼185 and ∼78 ppm), attributed to six-, seven- and eight-coordinated Y, respectively, and their relative intensities suggesting some preferential association of the vacancies with Zr rather than Y. A ^17^O MAS NMR spectrum of Y_2_Zr_2_O_7_ has also been acquired in previous work (for a sample enriched at 600 °C for 12 h),^27^ which contained a broadened lineshape proposed to result from the overlap of lines from the five different O species, all of which were thought to have small *C*_Q_ values, although these were not resolved directly.

**Fig. 6:**
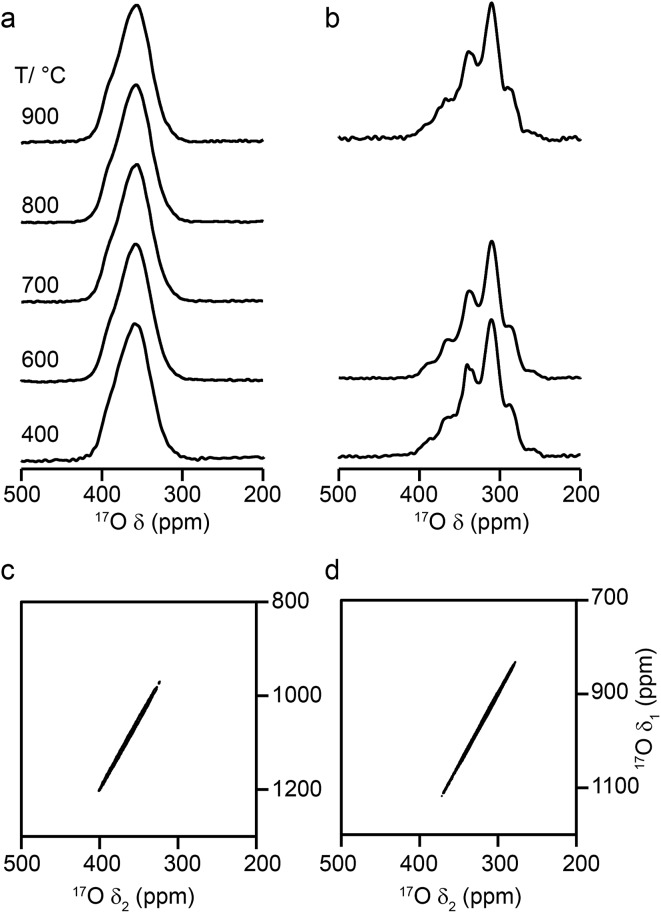
^17^O (14.1 T, 21 kHz) MAS NMR spectra of (a) Y_2_Zr_2_O_7_ and (b) Y_2_Hf_2_O_7_ enriched at temperatures between 400 and 900 °C for 24 h. (c, d) ^17^O (20.0 T, 21 kHz) triple-quantum MAS NMR spectra, acquired using a z-filtered pulse sequence, of (c) Y_2_Zr_2_O_7_ and (d) Y_2_Hf_2_O_7_ enriched at 950 °C for (c) 24 h and (d) 48 h.

Fig. 6 shows ^17^O MAS NMR spectra of Y_2_Zr_2_O_7_ and Y_2_Hf_2_O_7_, enriched at temperatures between 400 and 900 °C for 24 h. As discussed in the ESI (S1†), there were no significant changes to the ^89^Y NMR spectra after enrichment. For Y_2_Zr_2_O_7_, a broadened resonance is observed centered at ∼360 ppm, in good agreement with previous work.^27^ The ^17^O MQMAS spectrum (Fig. 6d) confirms all species are subject to very small quadrupolar interactions (*P*_Q_ values of ∼1–1.5 MHz), and that the broadening observed arises from a distribution of chemical shifts (as a result of the lack of long-range order). Very little difference is seen in the spectral lineshape as the enrichment temperature is varied. Although not shown directly in Fig. 6 (where spectra were acquired with different numbers of transients), the overall level of enrichment is considerably lower (by a factor of ∼6) for the sample enriched at 400 °C, and no signal was observed for sample enriched at temperatures any lower than this. Little difference in absolute intensity was observed with enrichment temperatures above 600 °C. For Y_2_Hf_2_O_7_, signal is observed at lower chemical shift, and a number of individual resonances are resolved. The ^17^O MQMAS spectrum (Fig. 6c) again confirms little evidence for any significant quadrupolar broadening (all *P*_Q_ values of ∼1–1.5 MHz). As observed for Y_2_Zr_2_O_7_, there is very little difference in the spectral lineshape as the enrichment temperature is varied, but for samples enriched at 400 °C there was a significant decrease in the overall signal intensity. There was also little difference observed in the relative intensities of the components in the spectrum with enrichment time see ESI (Fig. S9.1†).

Although a number of components are observed in the ^17^O MAS NMR lineshape of Y_2_Hf_2_O_7_, it is difficult to assign these to specific chemical environments. Six resonances are present (at ∼387 (2%), ∼364 (11%), ∼337 (29%), ∼310 (46%), ∼288 (11%) and ∼260 (1%) ppm). The systematic shift (of ∼23–27 ppm) between these components might reasonably be attributed to the systematic substitution of Hf for Y in the local coordination environment. However, only five such local environments (OY_4_, OY_3_Hf, OY_2_Hf_2_, OYHf_3_ and OHf_4_) would be expected, and six resonances are observed (albeit two with low intensity). A range of additional experiments (described in detail in the ESI (S9†)) confirm that: (i) the lineshapes observed do not result from the presence of significant quadrupolar broadening; (ii) all six signals result from CT rather than ST; (iii) none of the six signals result from impurity phases (although the presence of three low level impurities can be detected); (iv) there is no evidence for any paramagnetic impurity and/or paramagnetic shifts and (v) no signals can be confirmed as resulting from surface –OH species.

Some insight into possible assignments of the six resonances in the ^17^O NMR spectrum of Y_2_Hf_2_O_7_ can be obtained by comparing this to spectra of Y_2_Zr_2_O_7_, Y_2_Sn_2_O_7_ and Y_2_Ti_2_O_7_, all of which are expected to contain an OY_4_ environment spectra are shown overlaid in the ESI (Fig. S9.5†). The two pyrochlores have OY_4_ signals at 384 and 386 ppm, in good agreement with the resonance observed at ∼387 ppm for Y_2_Hf_2_O_7_, suggesting this could be assigned to this type of environment. The composite lineshape seen for Y_2_Zr_2_O_7_ also covers this frequency range. Substitution of Zr for Y would be expected to reduce the ionicity of the M–O bond (owing to the increased electronegativity of Zr (1.33) over Y (1.22)), resulting in an upfield shift of the signal. The similar, but slightly lower, electronegativity of Hf (1.3) has a similar effect on the signal. This leads to a tentative assignment of resonances at ∼364, ∼337, ∼310 and ∼288 ppm to OY_3_Hf, OY_2_Hf_2_, OYHf_3_ and OHf_4_ environments, respectively, leaving the peak at ∼260 ppm unassigned.

In recent years, there has been growing interest in complementing experimental measurement with theoretical calculations. Although is becoming almost routine for ordered solids, calculations are also increasingly being applied to help to understand and assign spectra of disordered systems, including ceramics, silicates and microporous frameworks.^56^–^58^ While it can be possible to gain some insight into the spectral assignment for systems exhibiting compositional disorder by predicting the effect of changes to the local environment in a systematic way, this becomes progressively more difficult as the level and types of disorder present increases. In principle, it is possible to calculate the total number of possible arrangements of a fixed number of atoms (or vacancies) on a fixed number of crystallographic sites using simple statistics. In the defect fluorite materials, if we consider a “unit cell” of similar size to that of a pyrochlore (*i.e.*, a 2 × 2 × 2 supercell of the defect fluorite structure), there are 32 cation sites (occupied by 16 Y and 16 Zr or Hf) and 64 anion sites (occupied by 56 oxygens and 8 vacancies). This leads to a possible 2.64 × 10^18^ possible structures (∼600 million possible cation arrangements and ∼4.4 billion possible anion arrangements). Although many of these will be related by symmetry, reducing the number of unique arrangements that would need to be considered, it is clearly not feasible to consider all of these and a subset must be selected to give some insight into the NMR parameters and determine whether it is possible to assign the resonances seen in the spectrum.

**Fig. 7:**
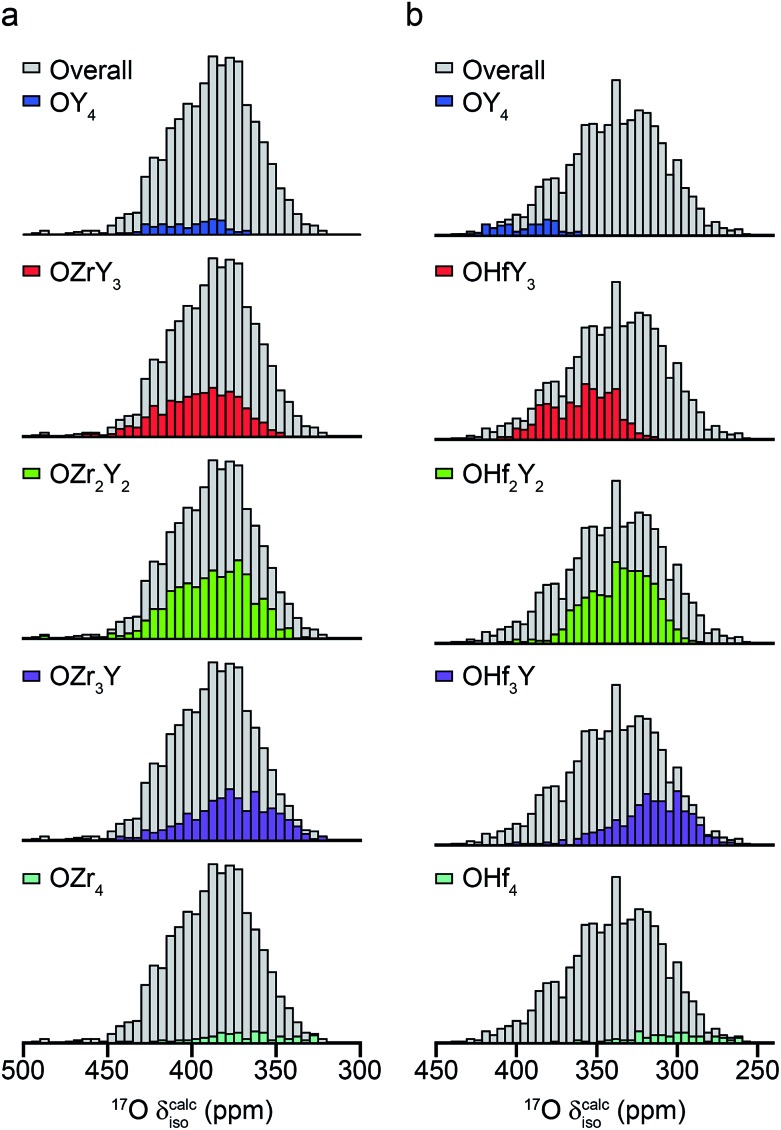
Calculated ^17^O chemical shifts for ∼30 structural models of (a) Y_2_Zr_2_O_7_ and (b) Y_2_Hf_2_O_7_, separated by local atomic environment. Points corresponding to the four additional pyrochlore-based structural models (described in detail in the ESI (S10†)) are not included.

The ESI (S10†) describes how ∼34 structural models (for each of Y_2_Zr_2_O_7_ and Y_2_Zr_2_O_7_) were selected, and the process undertaken to predict NMR parameters. The chemical shifts and *C*_Q_ values predicted are shown in the ESI (S10†), and the former also plotted as histograms in Fig. 7. For Y_2_Zr_2_O_7_, there is a small decrease in the average chemical shift as the number of Zr neighbours increases, but there is significant overlap of the range of shifts predicted for different environments. This is in good agreement with the experimental spectrum, where resonances from O species with different local environments are not resolved, and the lineshape observed covers ∼100 ppm. As discussed in the ESI (S10†), these models span an energy range of 5.35 eV, with the most stable 0.41 eV lower in energy than Y_2_Zr_2_O_7_ ordered pyrochlore. Notably, this pyrochlore structure has similar ^17^O shifts to those from the disordered models (as shown in the ESI (S10†)), while the shifts predicted for a reverse Y_2_Zr_2_O_7_ pyrochlore (*i.e.*, with Zr on the 8 coordinate A site and Y occupying the six-coordinate B site) are at the more extreme ends of the ranges observed (and this structure is significantly higher in energy). Even with the small number of structural models generated, differences are apparent for Y_2_Hf_2_O_7_ – as shown in Fig. 7, there is a more significant change in the average isotropic shift (of 20 to 30 ppm upfield) with each additional Hf bonded to O, in good agreement with the separation of the resonances observed in the experimental spectrum. However, for each environment type a broad range of shifts is again predicted, as shown in the histogram plotted in Fig. 7b, suggesting that the spectral resonances observed experimentally may well result from the overlap of signals from different environments (as for Y_2_Zr_2_O_7_, calculated values of *C*_Q_ are similar for each type of environment, as shown in the ESI (S10†)). Therefore, although it is tempting to assign the apparently well-resolved resonances (and subsequently comment on the cation clustering/ordering in this material), the computational results suggest that a definitive spectral assignment is not possible at this stage, and this will be the subject of a more detailed future multinuclear experimental and computational study.

### Layered perovskite-like materials

**Fig. 8:**
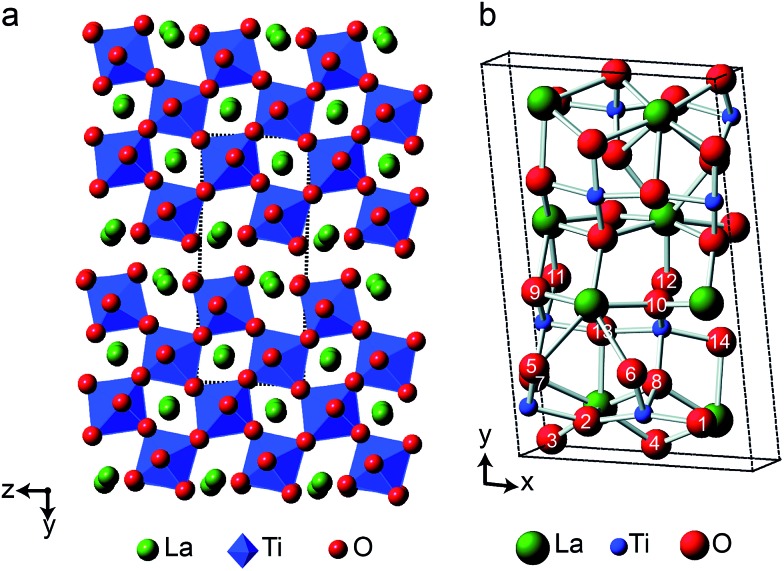
(a) Structure of layered perovskite La_2_Ti_2_O_7_, viewed down the (100) axis. Red spheres denote O, green spheres denote La cations, and TiO_6_ octahedra are shown in blue. The dashed lines indicate the monoclinic unit cell. (b) Monoclinic unit cell of La_2_Ti_2_O_7_ viewed down the (001) axis, with the crystallographically distinct O species indicated.

As the relative difference in size between the cations increases, and *r*_A_/*r*_B_ exceeds 1.78, A_2_B_2_O_7_ phases adopt a layered perovskite-like structure, as shown in Fig. 8.^23^,^24^ For La_2_Ti_2_O_7_, there has been some debate over the exact structure of this phase, with two suggested forms at room temperature; monoclinic (*P*2_1_)^59^ and orthorhombic (*Pna*2_1_).^60^ The two proposed models are similar, but differ in the length of the *c* axis, which is twice as large in the orthorhombic structure. A range of more recent work^61^,^62^ has confirmed a monoclinic structure is observed, but it is not completely clear whether an orthorhombic modification does exist at ambient temperatures, or whether this structure solution was the result of crystal twinning. Recent ^119^Sn NMR studies of Sn-doped La_2_Ti_2_O_7_ suggested preferential substitution of Sn onto just two of the four possible Ti sites (*i.e.*, into those in the bulk, rather than on the edge of the perovskite layers), but DFT calculations predicted very similar ^119^Sn chemical shifts for the two models and did not distinguish between them.^35^ La_2_Ti_2_O_7_ itself was not part of this prior study, as ^139^La and ^47/49^Ti are challenging nuclides to study, particularly in disordered materials. In principle, ^17^O NMR spectroscopy could provide further insight and, ultimately, may offer an additional opportunity to explore cation substitution in these layered perovskite ceramics.

**Fig. 9:**
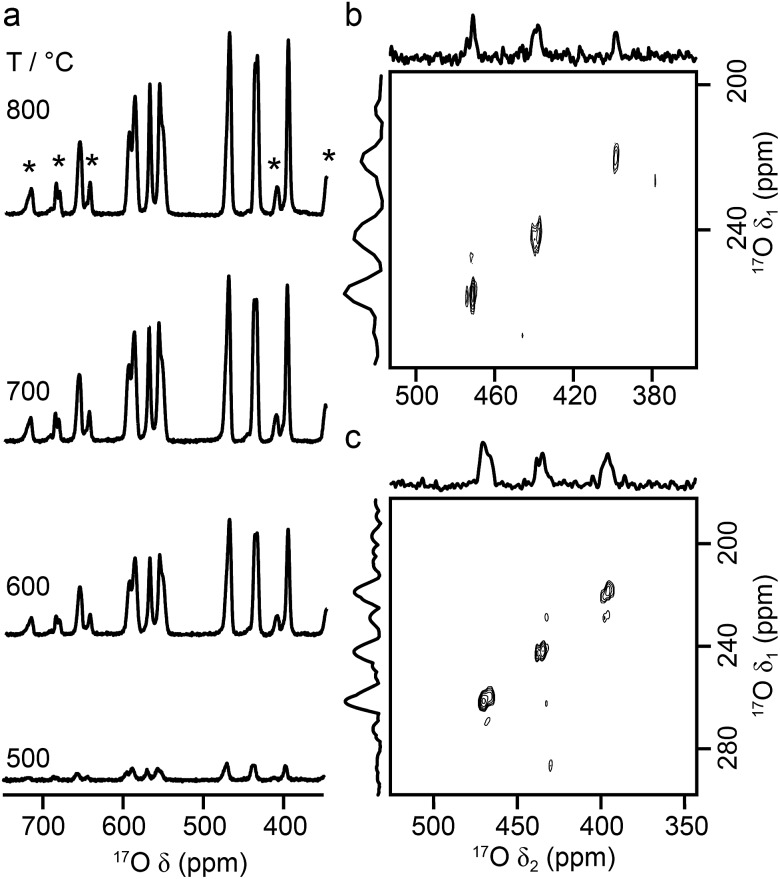
(a) ^17^O (14.1 T, 21 kHz) MAS NMR spectra of La_2_Ti_2_O_7_ enriched for 12 h at temperatures between 500 and 800 °C. (b, c) ^17^O triple-quantum MAS NMR spectra, acquired using a z-filtered pulse sequence (and shown after a shearing transformation), of La_2_Ti_2_O_7_ enriched at 950 °C for 48 h, acquired at (b) 14.1 T with 20 kHz MAS and (c) 9.4 T with 13.5 kHz MAS.

Fig. 9a shows ^17^O MAS NMR spectra of La_2_Ti_2_O_7_ enriched at different temperatures for 12 h. When enrichment is carried out at 500 °C there is a low level of absolute enrichment, but above 500 °C there is very little change in the relative intensities of the peaks in the spectrum (although the absolute level of enrichment changes with temperature and time – see ESI (S11†)). There are 14 distinct oxygen sites expected, but only 7 resonances are observed (as it is unclear whether any of the shoulders and/or splittings seen result from quadrupolar broadening or the overlap of resonances from distinct sites). No difference is seen in the relative intensities of the resonances, either as a function of enrichment temperature or enrichment time. Integration of the lineshapes gives relative intensities of 1.1 : 2.9 : 1.4 : 2.4 : 2.8 : 2.8 : 2.0 (in order of decreasing shift), and this does not vary with any increase in recycle interval. However, it is difficult to say if this represents quantitative enrichment without understanding the contribution of the quadrupolar interaction to the lineshapes, any difference in the nutation rates of each species, and whether there is any contribution from the STs. No additional resonances are clearly resolved in spectra acquired at higher *B*_0_ field strength (not shown), and there are only very small changes observed in the positions of the signals, suggesting *C*_Q_ values are small for all species. ^17^O MQMAS spectra (acquired at 14.1 T and 9.4 T), shown in Fig. 9b and c, contain only three signals, all below 480 ppm, suggesting only these resonances have any significant quadrupolar coupling (the efficiency of triple-quantum filtration falls sharply to zero as *C*_Q_ decreases). *P*_Q_ values for the observed resonances are between ∼1 and ∼2 MHz.

**Fig. 10:**
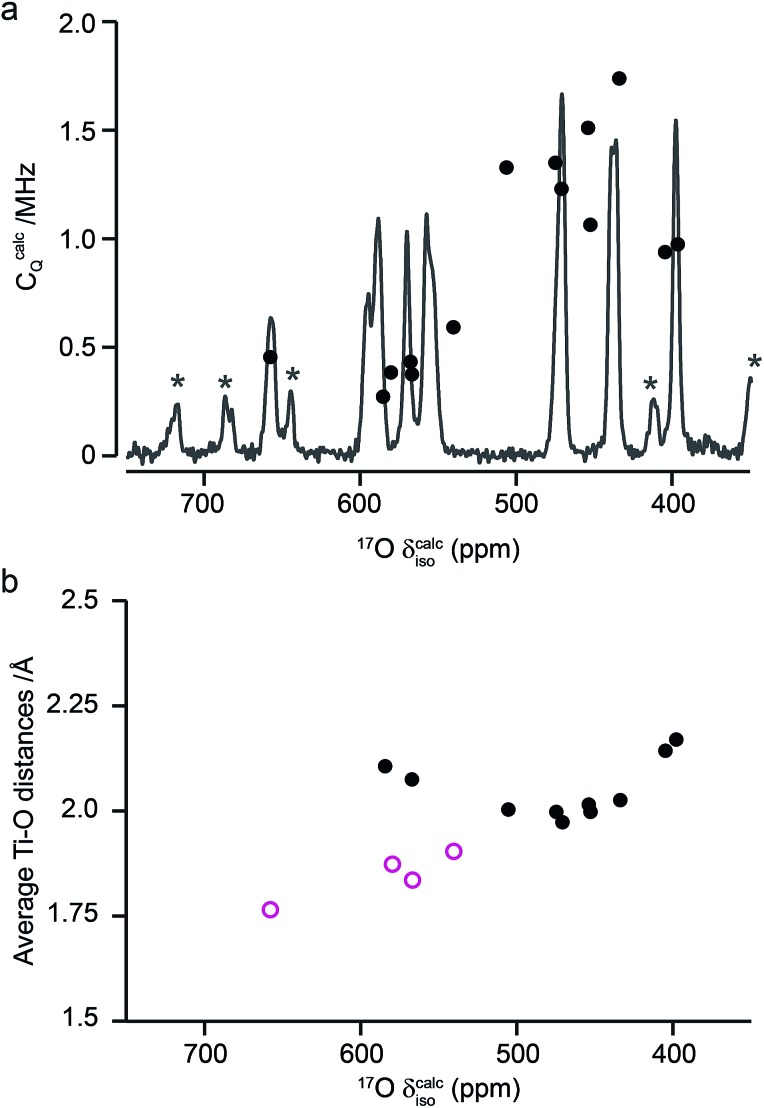
(a) Calculated NMR parameters (*δ*calciso and *C*calcQ) for the monoclinic structural model of La_2_Ti_2_O_7_. Also shown is the ^17^O (14.1 T) MAS NMR spectrum of La_2_Ti_2_O_7_ enriched at 900 °C for 12 h. Note the position of the resonances in the experimental spectrum will, in principle, result from both the isotropic chemical shift and any isotropic quadrupolar shift. (b) Plot of average O–Ti distance against *δ*calciso. O species with just one bond to Ti are shown as pink open circles.

Fig. 10a compares the isotropic chemical shifts (*δ*calciso) calculated by DFT for the monoclinic structural model of La_2_Ti_2_O_7_ to the position of the lines in the experimental ^17^O MAS NMR spectrum (note that a different reference was used for this calculation, as explained in detail in the ESI (S12†)). Also shown are the calculated quadrupolar coupling constants, *C*calcQ. Exact calculated NMR parameters for all 14 O species are given in the ESI (Table S12.1†). The calculated parameters are in reasonable agreement with experiment, with the resonances falling into three groups; one O (O10) at *δ* above 660 ppm, 5 O between 550 and 600 ppm, and 8 O below 500 ppm. The *C*_Q_ values predicted for these latter species are larger than the other six, in agreement with the ^17^O MQMAS spectra shown in Fig. 9. At 14.1 T, this would result in almost negligible isotropic quadrupolar shifts of only 0.1 to 2.7 ppm, within the typical linewidths observed. It should be noted (as discussed in the ESI (S12†)) that the predicted NMR parameters are very similar for the proposed orthorhombic structure of La_2_Ti_2_O_7_, and it is not possible to distinguish between these using ^17^O NMR spectroscopy.

As seen in Fig. 10a, a complete and unambiguous spectral assignment is not possible. However, considering the relative intensities of the resonances in the experimental spectrum, and the magnitude of the isotropic chemical shifts predicted by DFT, a partial spectrum assignment can be obtained, as shown in Table 3. This assignment would result in ideal relative spectral intensities of 1.0  :  2.0 : 1.0 : 2.0 : 3.0 : 3.0 : 2.0. While this is in reasonable agreement with experiment, the largest differences are seen for the group of signals between 597 and 554 ppm (which should integrate to 5, rather than 6, O). However, as can be seen from Fig. 10a, these are the signals predicted to have the smallest *C*_Q_ values and, therefore, the largest contributions from their STs. These can be estimated using density matrix simulations, and lead to corrected experimental intensities of (approximately) 1.0 : 2.1 : 1.2 : 2.1 : 3.0 : 3.0 : 1.9, in good agreement with theoretical predictions, confirming the (partial) assignment in Table 3. This result also indicates that the isotopic enrichment is quantitative for the 14 oxygen sites in this material for samples heated at and above 600 °C for 12 h.

**Table 3:** Partial assignment of the ^17^O MAS NMR spectrum of La_2_Ti_2_O_7_ shown in Fig. 10a

*δ* (ppm)	Assignment
658	O10
597	(O6 or O11)
589	(O6 or O11)
571	(O5 or O9)
558–554	(O5 or O9) + O12
471	O14 + O8 + O7
438	O13 + O1 + O2
398	O3 + O4

The ^17^O chemical shifts in La_2_Ti_2_O_7_ are spread over ∼250 ppm, suggesting significant differences in local environments. However, the number and type of coordinating atoms are difficult to define, as this depends upon the exact distance around the central atom that is considered (as seen in Table S12.1 of the ESI†). It is clear that the species with just one bonded Ti (*i.e.*, O9–12) have some of the highest shifts present, leading to the suggestion the O–Ti distance may be the dominant influence on *δ*_iso_. Fig. 10b plots the average O–Ti distance against isotropic chemical shift for the 14 oxygen species and shows there is reasonable positive correlation. The two species that lie just above the line correspond to O5 and O6, which have one long and one short O–Ti bond. In general, the species at the edge of perovskite layers (as shown in Fig. 8b) have the higher shifts, while those in the bulk (*i.e.*, O1–4) tend to have lower isotropic chemical shifts.

## Conclusions

In this work we have investigated the post-synthetic ^17^O enrichment of A_2_B_2_O_7_ ceramic oxides. For materials adopting a pyrochlore structure, enrichment was clearly non quantitative at lower temperatures (∼700 °C and below) and at shorter times, with preferential enrichment of the O2 (OA_2_B_2_) environment. Only at higher enrichment temperatures (or for longer durations at lower temperatures) were consistent relative spectral intensities achieved, an observation of considerable future importance for using ^17^O NMR spectroscopy to study cation disorder in solid solutions. There was also a significant difference in the rate of relative enrichment between the different pyrochlores. Although this might reflect the variation in bond dissociation energies, previous computational work^53^–^55^ has indicated that the pathway for ion diffusion is similar for La_2_Sn_2_O_7_ and Y_2_Ti_2_O_7_ but different for Y_2_Sn_2_O_7_, and that the activation energies for oxygen ion migration are similar for the two stannate pyrochlores, but higher for Y_2_Ti_2_O_7_. Detailed conclusions are, therefore, difficult, but it is clear that enrichment at higher temperature (or potentially longer time) will be vital when using ^17^O NMR to study disordered materials or phase mixtures, to minimise any preferential/selective enrichment where quantitative results are required.

Although consistent relative spectral intensities were obtained for the pyrochlore materials at higher enrichment temperatures, these did not correspond to the 1 : 6 ratio expected from the crystal structure, suggesting that spectral acquisition itself was not quantitative. At the short flip angles used, nutation differences were shown not to be a problem. Even after correcting for measured differences in *T*_1_ relaxation rates, the expected intensity ratio was not observed. This was shown to be a result of the additional need to also correct for the contribution of the STs to the two resonances (a particularly large contribution for the O1 sites, which have very small *C*_Q_). While this is perhaps of most importance for the (ordered) end-member pyrochlores, if differences in the magnitude of *C*_Q_ are still present, these corrections may still need to be taken into account for disordered materials.

For the defect fluorite phases the spectral lineshapes (and therefore the rates of relative enrichment) change little with enrichment temperature or time, although an increase in the absolute level of enrichment was observed at the higher temperatures. For Y_2_Zr_2_O_7_, a complex, overlapped lineshape was observed, while for Y_2_Hf_2_O_7_, the individual components were better resolved, reflecting the greater difference in the ionicities. In the latter case, however, the spectrum appeared to consist of six (rather than the expected five) resonances. DFT calculations for a series of disordered models suggest each signal may have contributions from a number of different chemical environments, and that it might not be possible to assign the signals unambiguously and use relative intensities to provide insight into disorder. This complex problem will form the basis of further future investigation.

The layered perovskite-like structure adopted by La_2_Ti_2_O_7_ results in 14 distinct O species, although these were not all resolved using ^17^O NMR spectroscopy even at high field. Comparison to DFT calculations enabled a partial assignment of the spectrum and demonstrated the similarity of some of the O species. This also enabled accurate integrated intensities (after correction for the contributions of the STs) to be determined, demonstrating that quantitative enrichment was achieved. There was relatively little difference in the rate of enrichment of the distinct sites with enrichment temperature above 600 °C, although the highest absolute levels of enrichment were obtained at the highest temperatures.

Our results suggest that ^17^O NMR spectroscopy could provide a useful probe of disorder in ceramic materials, and will be of particular use when the materials studied do not possess other nuclei with “favourable” NMR properties. However, we have demonstrated that, if quantitative results are required, it is necessary (i) to pay close attention to the temperatures and times used for post-synthetic gas exchange to mitigate any preferential or selective enrichment and (ii) to choose the experimental acquisition parameters carefully, correcting for differences in relaxation and the contribution of ST to the spectral resonances observed. This work provides a vital step in exploiting ^17^O NMR spectroscopy for the quantitative study of compositional and positional disorder in ceramics, and the study of multi-phase materials. With careful consideration of synthetic and spectroscopic parameters ^17^O NMR spectroscopy has the potential to play a vital role in the structural characterisation of complex oxide ceramics.

## Conflicts of interest

There are no conflicts to declare.

## Supplementary Material

Supplementary information
