# Supplementary material for: 17O solid-state NMR spectroscopy of A2B2O7 oxides: quantitative isotopic enrichment and spectral acquisition?
Source: RSC Adv. 2018 Feb 14;8(13):7089–101. doi: 10.1039/c8ra00596f (PMC5815285; doi:10.1039/c8ra00596f)
Supplement: RA-008-C8RA00596F-s001 [file RA-008-C8RA00596F-s001.pdf]

Electronic Supplementary Material (ESI) for RSC Advances.
This journal is © The Royal Society of Chemistry 2018

^{17}O Solid-State NMR Spectroscopy of Ceramic Oxides: Quantitative Isotopic Enrichment and Spectral Acquisition?

Arantxa Fernandes, Robert F. Moran, Scott Sneddon, Daniel M. Dawson, David McKay, Giulia P. M. Bignami, Frédéric Blanc, Karl R. Whittle and Sharon E. Ashbrook

Supporting Information

S1. Powder XRD patterns, SEM images and mass spectrometry

S2. ^{89}Y NMR spectra acquired pre and post ^{17}O enrichment

S3. Referencing of ^{17}O DFT calculations

S4. ^{17}O slow MAS spectra

S5. NMR of $La_2Sn_2O_7$

S6. NMR of $Y_2Ti_2O_7$ and $La_2Sn_2O_7$ enriched under different conditions

S7. Nutation simulations and correction to spectral intensities

S8. Overall levels of ^{17}O enrichment

S9. Additional ^{17}O NMR spectra of $Y_2Hf_2O_7$ and impurity phases

S10. DFT calculations for $Y_2Zr_2O_7$ and $Y_2Hf_2O_7$

S11. ^{17}O NMR of $La_2Ti_2O_7$

S12. DFT calculations for $La_2Ti_2O_7$

S13. References

S1. Powder XRD patterns, SEM images and mass spectrometry

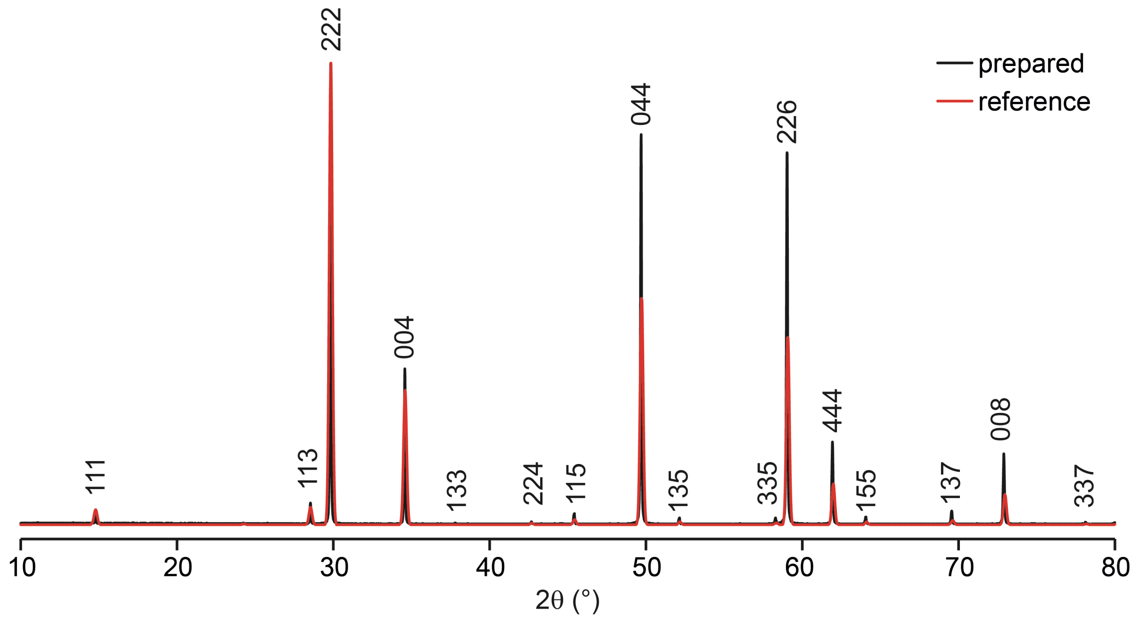

Figure S1.1 Typical experimental powder XRD pattern for a synthesised sample of $Y_2Sn_2O_7$ and powder pattern simulated from literature data (ICSD 157673). Peak intensities are normalised relative to the 222 peak.

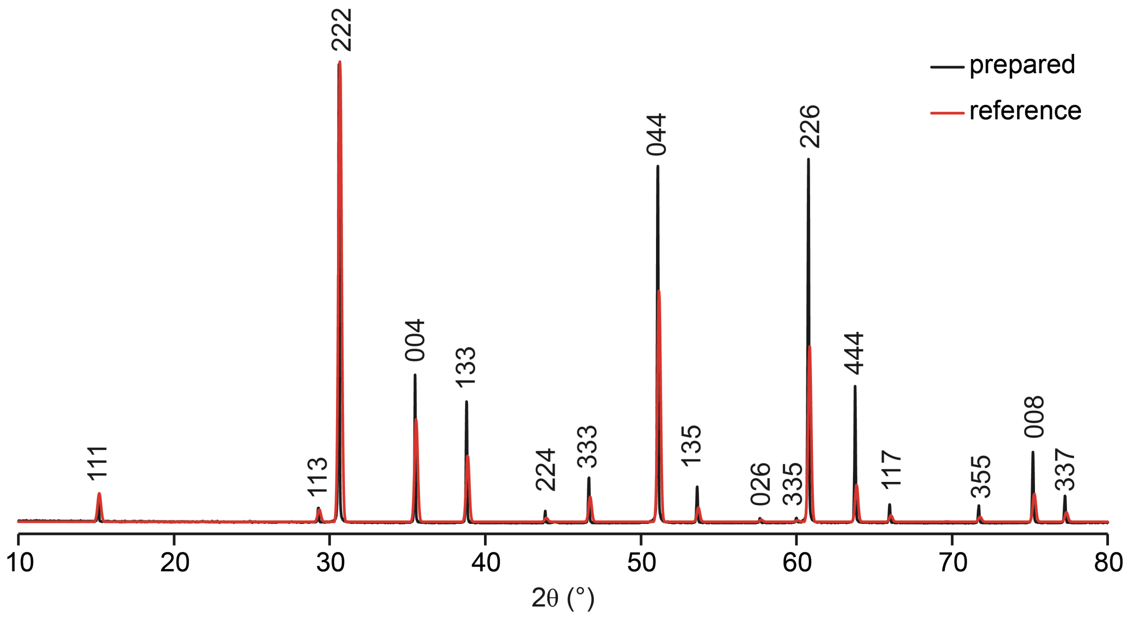

Figure S1.2 Typical experimental powder XRD pattern for a synthesised sample of $Y_2Ti_2O_7$ and powder pattern simulated from literature data (ICSD 24206). Peak intensities are normalised relative to the 222 peak.

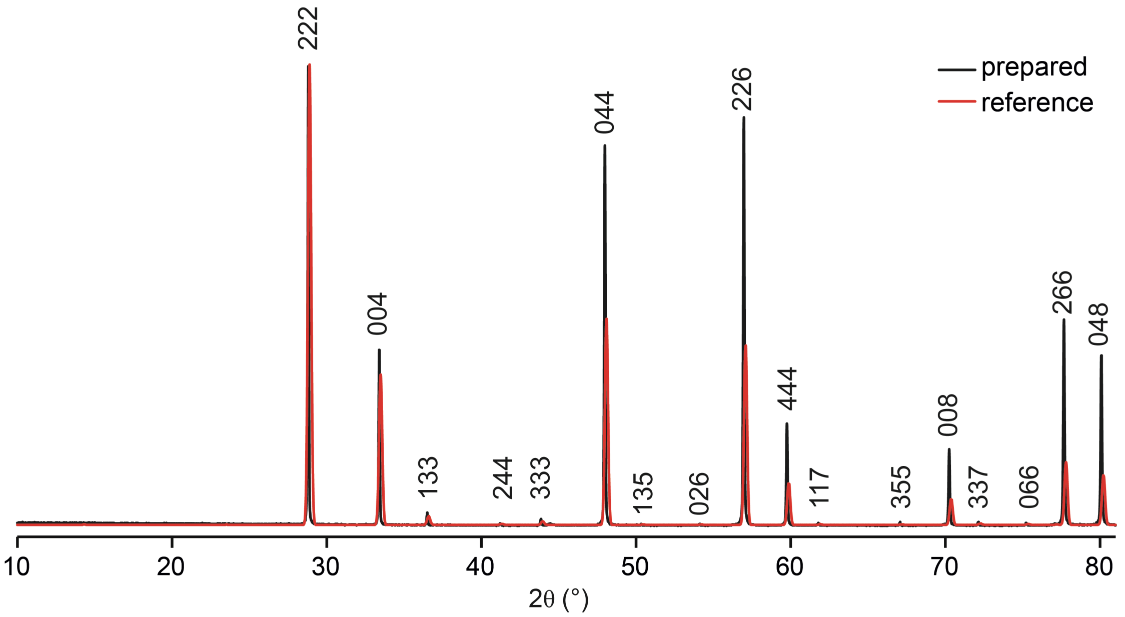

Figure S1.3 Typical experimental powder XRD pattern for a synthesised sample of $La_2Sn_2O_7$ and powder pattern simulated from literature data (ICSD 82956). Peak intensities are normalised relative to the 222 peak.

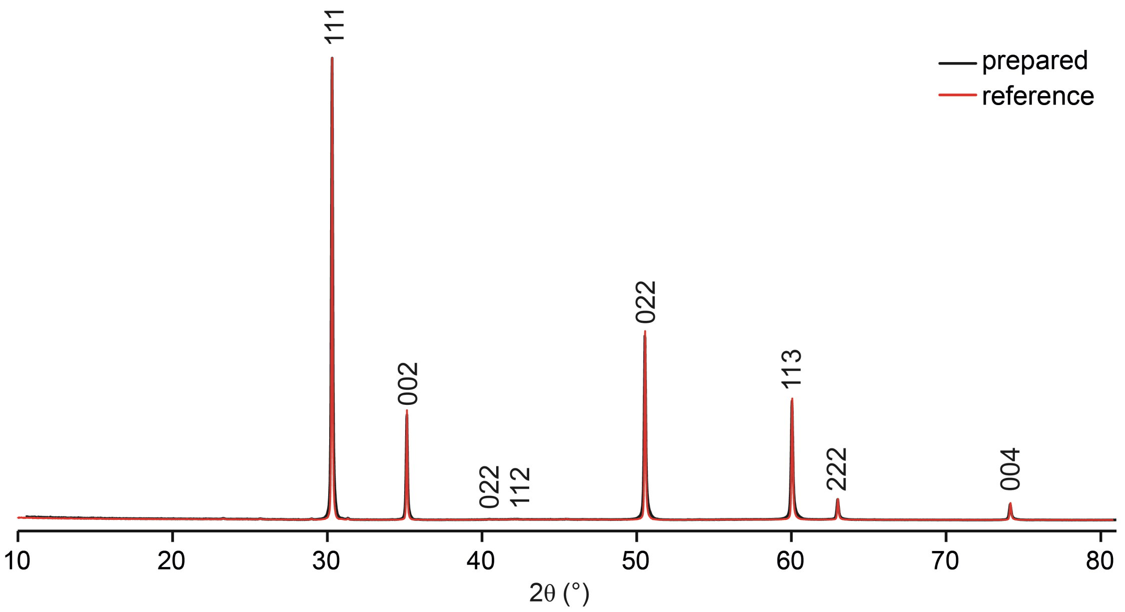

Figure S1.4 Typical experimental powder XRD pattern for a synthesised sample of $Y_2Zr_2O_7$ and powder pattern simulated from literature data (ICSD 153818). Peak intensities are normalised relative to the 111 peak.

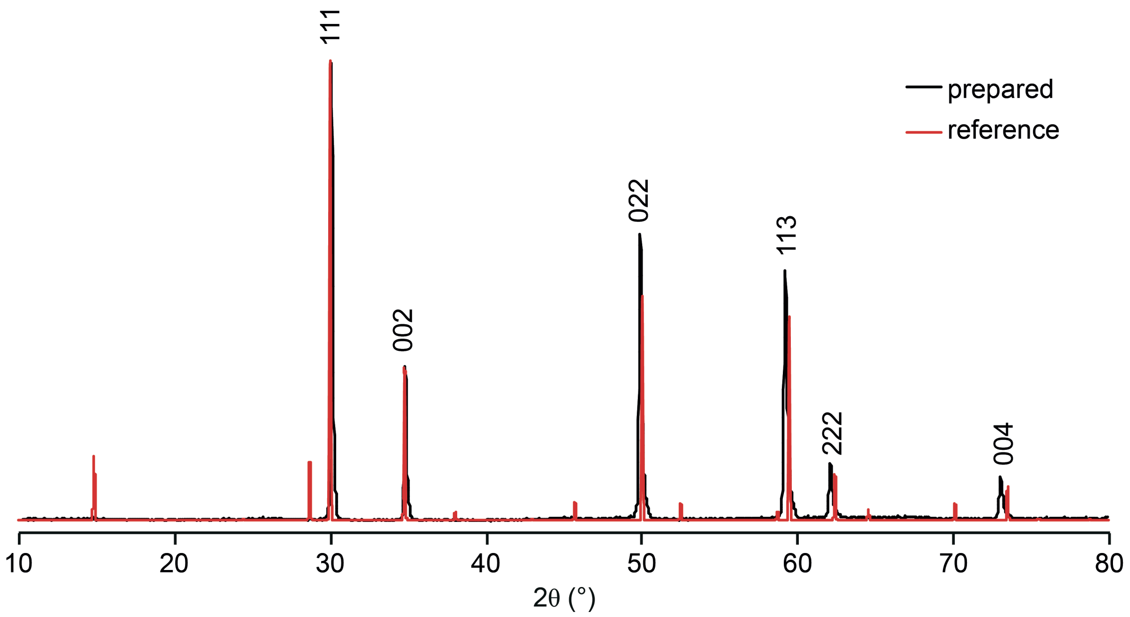

Figure S1.5 Typical experimental powder XRD pattern for a synthesised sample of $Y_2Hf_2O_7$ and powder pattern simulated from literature data (ICSD 153819). Peak intensities are normalised relative to the 111 peak.

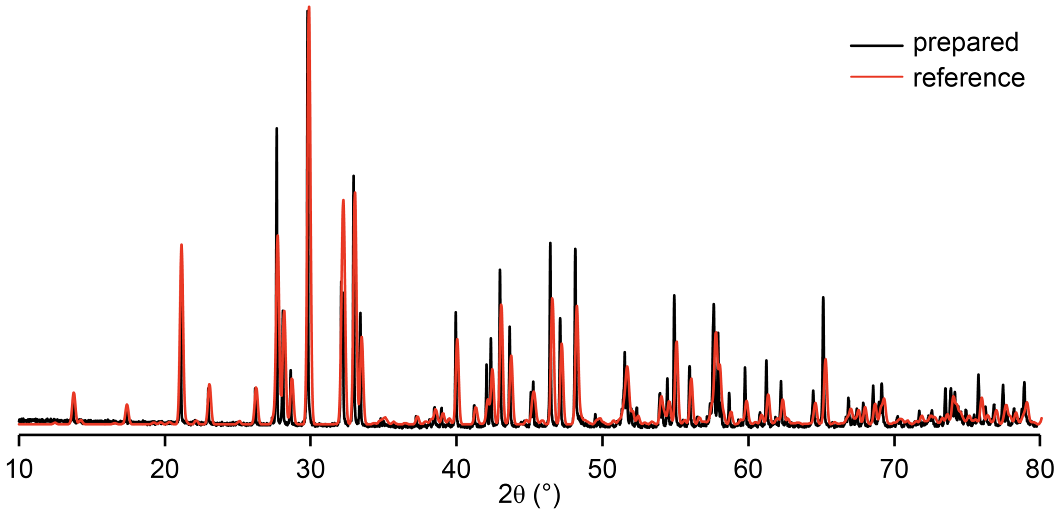

Figure S1.6 Typical experimental powder XRD pattern for a synthesised sample of $La_2Ti_2O_7$ and powder pattern simulated from literature data (ICSD 1950). Peak intensities are normalised relative to the peak at ~ 30° 2θ.

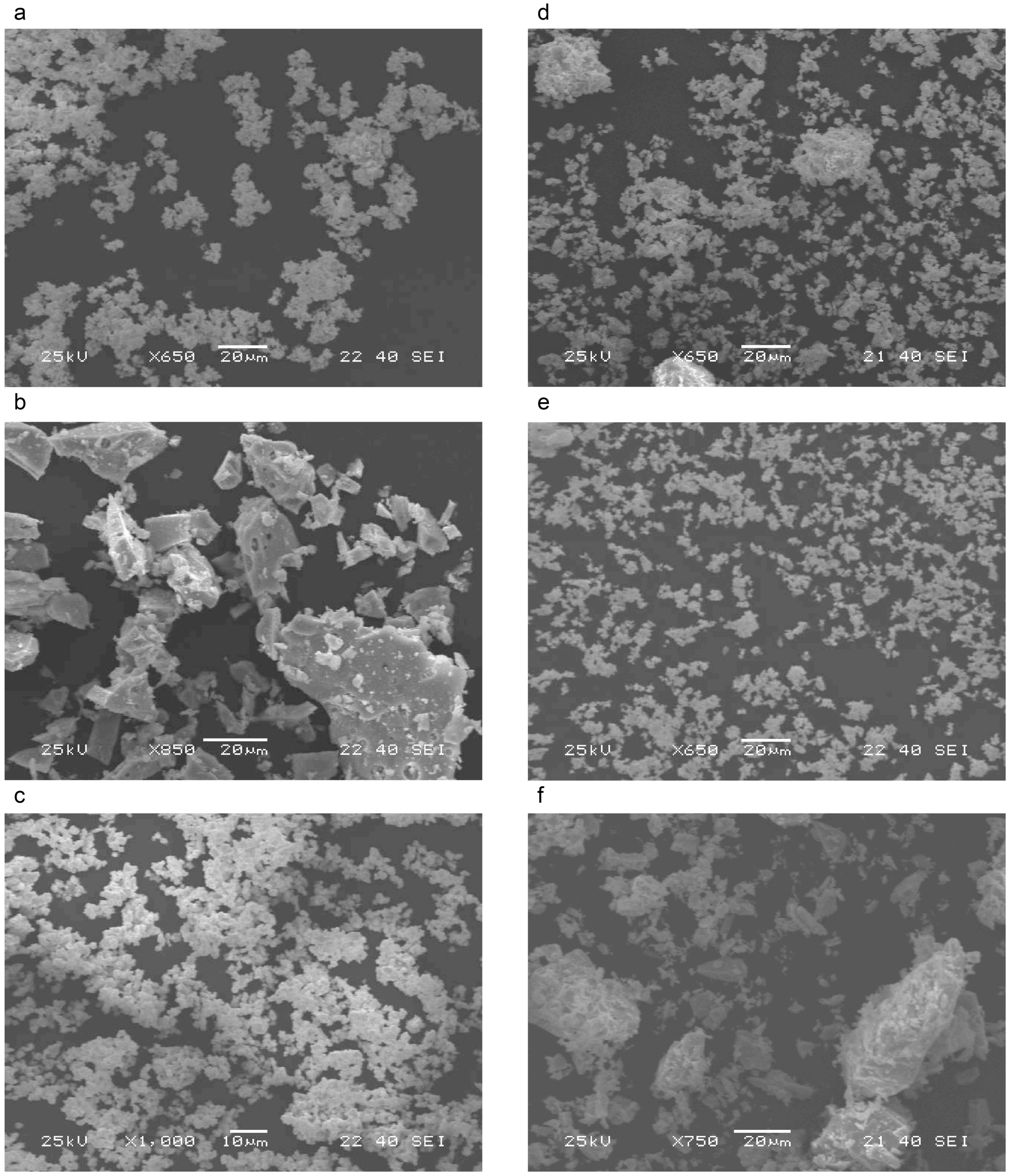

Figure S1.7 SEM images of powdered samples of (a) $Y_2Sn_2O_7$, (b) $Y_2Ti_2O_7$, (c) $La_2Sn_2O_7$, (d) $Y_2Zr_2O_7$, (e) $Y_2Hf_2O_7$ and (f) $La_2Ti_2O_7$.

Mass Spectrometry

Samples were studied using mass spectrometry, as described in the main text. Results are reported below as counts per isotope, with the instrumental standard error of the mean per measurement.

Table S1.1 O isotopes in $Y_2Sn_2O_7$ enriched at 900 °C for 12 h. The amount of ^{17}O detected is ~5.0%.

^{16}O (counts)	% standard error	^{17}O (counts)	% standard error	^{18}O (counts)	% standard error
5.95×10^5	0.43	3.1×10^4	0.75	2.59×10^3	0.64
2.08×10^6	0.21	1.06×10^5	0.30	9.11×10^3	0.25
1.24×10^6	0.39	7.01×10^4	0.51	5.65×10^3	0.40
1.37×10^6	0.37	7.32×10^4	0.28	6.00×10^3	0.32

Table S1.2 O isotopes in $Y_2Hf_2O_7$ enriched at 900 °C for 24 h. The amount of ^{17}O detected is ~7.5%.

^{16}O (counts)	% standard error	^{17}O (counts)	% standard error	^{18}O (counts)	% standard error
1.22×10^6	0.77	9.7×10^4	0.80	6.78×10^3	0.75
1.76×10^6	0.56	1.42×10^5	0.59	9.93×10^3	0.58
1.62×10^6	0.60	1.30×10^5	0.61	9.09×10^3	0.66
1.70×10^6	0.34	1.38×10^5	0.39	9.66×10^3	0.41

Table S1.3 O isotopes in ^{17}O-enriched $La_2Ti_2O_7$ enriched at 800 °C for 12 h. The amount of ^{17}O detected is ~13%.

^{16}O (counts)	% standard error	^{17}O (counts)	% standard error	^{18}O (counts)	% standard error
1.03×10^6	0.35	1.62×10^5	0.35	9.48×10^3	0.34
1.70×10^6	0.23	2.63×10^5	0.26	1.53×10^4	0.27
1.85×10^6	0.23	2.66×10^5	0.32	1.56×10^4	0.36
1.63×10^6	0.27	2.41×10^5	0.26	1.41×10^4	0.27

S2. ^{89}Y NMR spectra acquired pre and post ^{17}O enrichment

In order to ensure no significant structural changes had occurred during the enrichment process, powder XRD measurements (not shown) and ^{89}Y NMR spectra were acquired both pre and post enrichment. Figures S2.1 and S2.2 show ^{89}Y MAS NMR spectra (acquired at 14.1 T with a recycle interval of 5 s), of $Y_2Sn_2O_7$ and $Y_2Ti_2O_7$. (Note, the number of transients averaged was not kept constant). It is not possible to resolve J couplings to ^{17}O, owing to the broadening of the spectral lines.

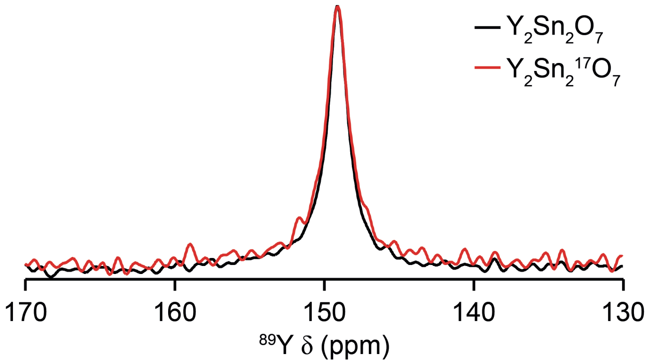

Figure S2.1 ^{89}Y (14.1 T, 14 kHz) MAS NMR spectra of $Y_2Sn_2O_7$ pre (black line) and post (red line) ^{17}O isotopic enrichment.

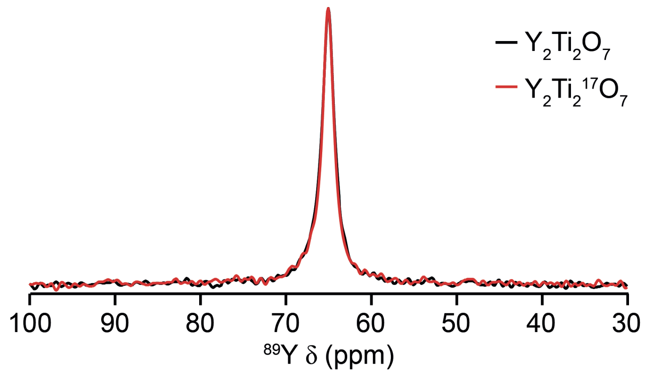

Figure S2.2 ^{89}Y (14.1 T, 14 kHz) MAS NMR spectra of $Y_2Ti_2O_7$ pre (black line) and post (red line) ^{17}O isotopic enrichment.

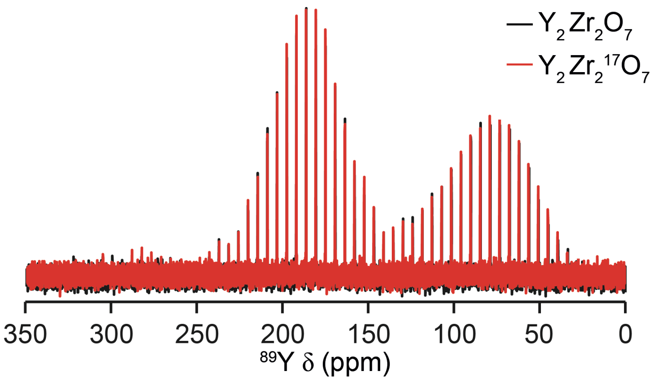

Figure S2.3 ^{89}Y (14.1 T, 14 kHz) CPMG MAS NMR spectra of $Y_2Zr_2O_7$ pre (black line) and post (red line) ^{17}O isotopic enrichment.

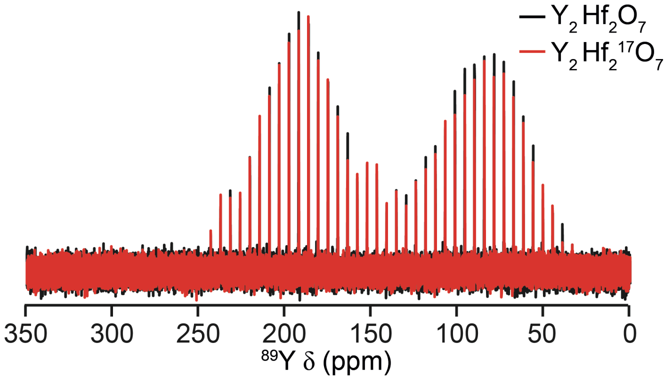

Figure S2.4 ^{89}Y (14.1 T, 14 kHz) CPMG MAS NMR spectra of $Y_2Hf_2O_7$ pre (black line) and post (red line) ^{17}O isotopic enrichment.

S3. Referencing of ^{17}O DFT calculations

In order to compare directly experimental chemical shifts to calculated values, the calculated isotropic chemical shielding values must be converted to a calculated shift by

$$\delta_{iso}^{calc} = \sigma_{ref} - \sigma_{iso}^{calc} \ , \qquad \text{(S3.1)}$$

where σ_{ref} is a reference shielding. The conventional way to determine σ_{ref} is to compare experimental shift and calculated shielding for a simple "model" compound where the experimental value is well determined and the structure well described. Even though this approach can work well, in some cases any uncertainty or error in either value (or any dependence of can lead to inaccuracies in referenced shifts for the materials of interest. If a material contains more than one distinct species, an alternative approach is to consider only the relative shift/shielding differences between the peaks (*i.e.*, determining a reference shielding from either one species or from the average shielding). A typically more robust approach, however, is to compare the experimental shift and calculated shielding for a much larger number of different types of systems that contain the nuclide of interest, thereby minimising any experimental errors or structural uncertainties.

Table S3.1 shows the calculated isotropic shieldings (σ_{iso}^{calc}) for a series of oxygen-containing materials and experimental isotropic shifts (δ_{iso}^{exp}) obtained from the literature, with the exception of $LiNbO_3$, which was measured in this work to resolve some ambiguity over literature parameters. In all cases, structural models were optimised prior to the calculation of NMR parameters, as described in the main text. Calculations were carried out using CASTEP version 8. Figure S3.1a plots δ_{iso}^{exp} against σ_{iso}^{calc} for all structures and shows a good linear correlation (R^2 = 0.9786). This was the procedure used to reference the calculations in the main text. However, the gradient of the line of best fit is not exactly equal to –1 (as would be expected for an ideal case), suggesting that the DFT calculations have a tendency to slightly overestimate any shielding differences. In this case a calculated shift value is produced using

$$\delta_{iso}^{calc} = (c - \sigma_{iso}^{calc})/m \ , \qquad \text{(S3.2)}$$

where c and m are the intercept and slope, respectively, from linear regression. In the ideal case, where m = 1, Eqn. S3.2 then simplifies to Eqn. S3.1 and c = σ_{ref}.

As shown in Figure S3.1b, a slightly better correlation (R^2 = 0.9896) is obtained if some of the simple oxides in Table S3.1 are not included in the analysis (those shown in red). Many of these compounds contain heavier nuclei (*i.e.*, those that might be more affected by relativity, which is only included in the ZORA approximation). It should also be noted that previous work has shown that the failure of GGA PBE to treat the unoccupied Ca 3d states correctly led to significant errors in the ^{17}O chemical shifts and CaO and calcium aluminosilicates.[S1] Calculations on the compounds highlighted in Table S3.1 may also suffer from similar problems. However, it should be noted that this results in a change to both m (now 0.9002) and c (now 228.808), leading to shielding values that can differ by 5-7 ppm at the extreme of the shielding range. This level of uncertainty may have relevance when attempting to assign some of the spectra discussed in the main text and suggests that the accuracy of the calculated values should always be treated with some caution. The equation given in Figure S3.1b was chosen to reference the calculations in the first parts of the main text.

Table S3.1 ^{17}O calculated chemical shielding (σ_{iso}^{calc}) and experimental chemical shifts (δ_{iso}^{exp}) for a range of inorganic compounds. The values for the structures shown in red were excluded from the plot in Figure S3.1b.

Compound	σ_{iso}^{calc} (ppm)	δ_{iso}^{exp} (ppm)
α-Cristobalite[S2]	220.72	37.2[S3]
α-Quartz[S4]	217.92	40.8[S3]
$MgSiO_3$ (perovskite)[S5]	129.17	108[S6]
$MgSiO_3$ (akimotoite)[S7]	135.37	106[S6]
α-Mg_2SiO_4[S8]	203.19	48[S9-11]
	184.78	64[S9-11]
	187.57	61[S9-11]
β-Mg_2SiO_4[S12]	214.38	38[S11,S13]
	169.41	76[S11,S13]
	180.29	66[S11,S13]
	182.35	65[S11,S13]
γ-Mg_2SiO_4[S14]	181.46	63[S11]
$SrTiO_3$ (perovskite)[S15]	–281.27	465[S16]
$BaZrO_3$ (perovskite)[S17]	–170.54	376[S16]
$LiNbO_3$[S18]	–222.50	449.9[b]
$LaAlO_3$[S19]	69.11	170.2[S16]
$Y_2Sn_2O_7$[S20]	–185.90	385[S21]
	28.37	165[S21]
$Y_2Ti_2O_7$[S22]	–172.17	385[S21]
	–257.48	455[S21]
Y_2O_3[S23]	–155.84	356[S24]
TiO_2 (rutile)[S25]	–398.38	591[S24,2S6]
$CaTiO_3$[S27]	–267.89	443.9[S16]
	–259.18	
$CaZrO_3$[S27]	–136.54	329.3[S16]

	–128.51	
BeO[S28]	231.67	26[S29]
MgO[S30]	197.80	47[S29]
CaO[S31]	–142.48	294[S29]
SrO[S32]	–206.25	390[S29]
BaO[S33]	–456.42	629[S29]
ZnO[S34]	174.60	–18[S29]
Bi_2O_3[S35]	–9.40	196[S24]
HgO[S36]	64.49	121[S29]
BaO_2[S37]	–58.65	334[S24]
La_2O_3[S38]	–304.92	582.4[S16]
	–194.38	466.5[S16]
HfO_2[S39]	–51.46	267[S40]
	–115.02	336[S40]
$MgSiO_3$ (clinoenstatite)[S41]	211.02	41[S42]
	203.64	45[S42]
	200.69	51[S42]
	196.17	54[S42]
	188.88	64[S42]
	176.40	75[S42]

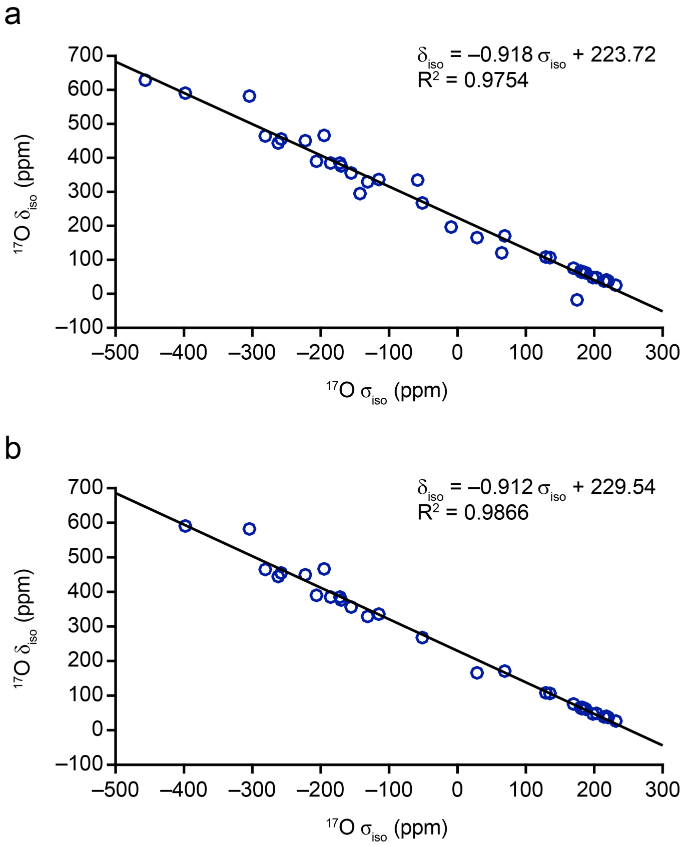

Figure S3.1 Plot of δ_{iso}^{exp} against σ_{iso}^{calc} for (a) all structures and (b) without those structures shown in red in Table S3.1.

S4. ^{17}O slow MAS spectra

Although, in principle, a C_Q value of zero is expected for O1 in ideal pyrochlore materials, spinning sidebands resulting from satellite transitions (ST) are observed in experimental spectra, indicating a small but non-zero magnitude for this interaction. The magnitude of the coupling constant can be estimated by fitting the sideband manifold in spectra acquired at slow (*i.e.*, 3.25, 3.6 and 6.5 kHz) MAS rates, as shown in Figure S4.1.

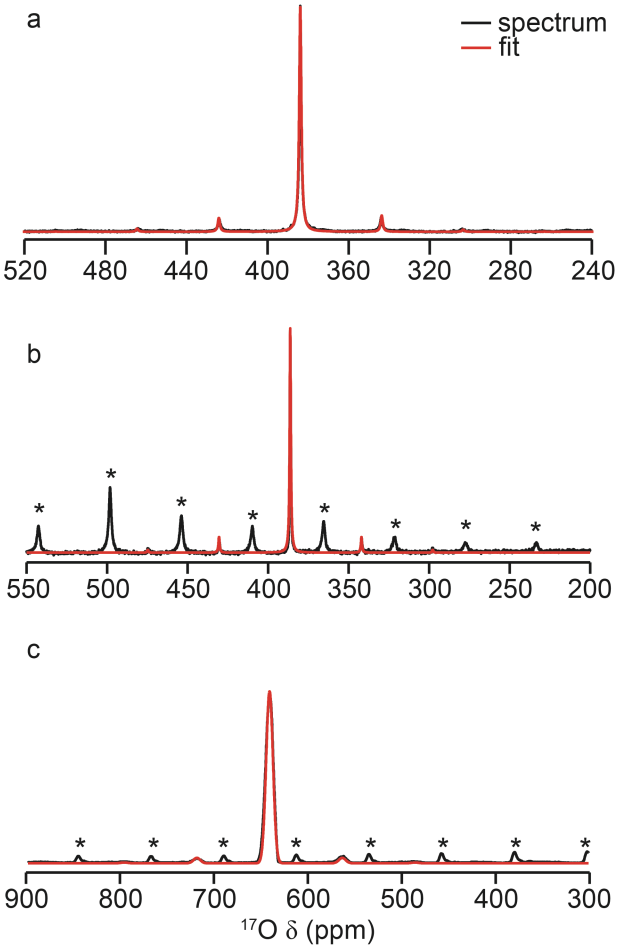

Figure S4.1 Expansion of sideband manifold resulting from the O1 species in the ^{17}O (14.1 T) MAS NMR spectra of (a) $Y_2Sn_2O_7$, (b) $Y_2Ti_2O_7$ and (c) $La_2Sn_2O_7$, along with the spectrum resulting from an analytical fit (red line). Spectra were acquired at (a) 3.25, (b) 3.6 and (c) 6.5 kHz MAS. Spinning sidebands resulting from O2 are marked *.

S5. NMR of $La_2Sn_2O_7$

The O1 resonance in $La_2Sn_2O_7$ exhibits a complex multiplet lineshape as a result of couplings to four ^{139}La neighbours. The lineshape is affected both by J couplings (constant value in Hz with field) and a quadrupolar-dipolar cross term (a second-order interaction that survives MAS).[S43-S44] Figure S5.1 shows the variation in this lineshape as a function of B_0 field strength.

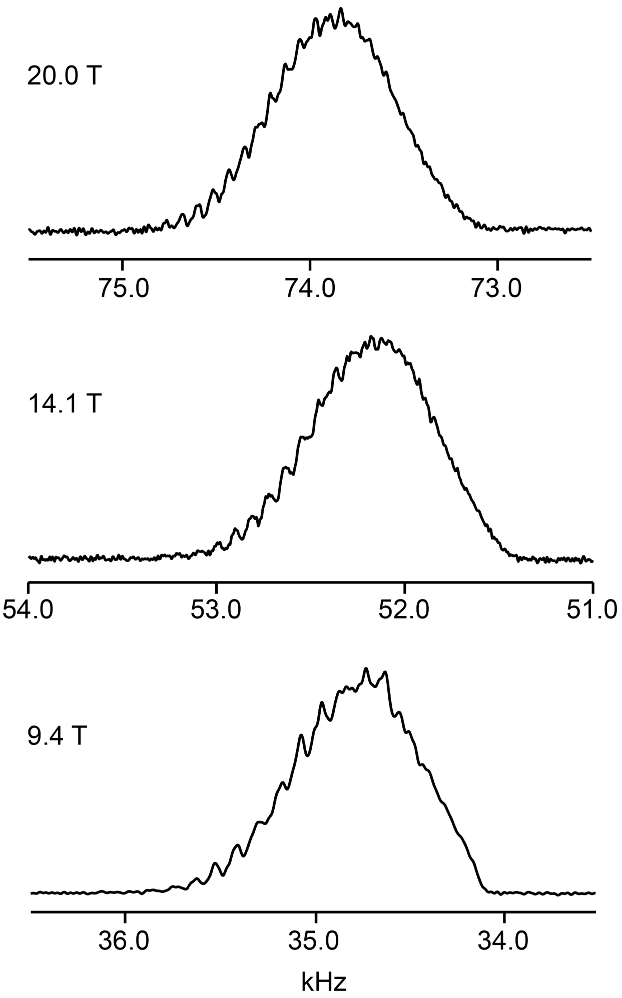

Figure S5.1 Expansion of lineshape corresponding to O1 species in ^{17}O MAS NMR spectra of $La_2Sn_2O_7$ at B_0 field strengths of 9.4, 14.1 and 20.0 T.

The magnitude of the quadrupolar-dipolar cross term depends on the ^{139}La C_Q, the magnitude of the $^{17}O/^{139}La$ dipolar coupling and the B_0 field at which the spectrum is acquired. DFT calculations predict ^{139}La C_Q = 76.4 MHz and η_Q = 0. These are in good agreement with the values measured experimentally ($C_Q \approx$ 79 MHz, $\eta_Q \approx$ 0) from the

frequency-stepped wideline CPMG spectrum shown in Figure S5.2. This fitting assumed the effect of the CSA on the broad quadrupolar lineshape to be negligible. This is supported by the spectra in Figure S5.3, simulated with and without the CSA included, using the NMR parameters predicted by DFT calculations. Given a O–La distance of 2.317 Å, this leads to estimates of the cross term between ~50 Hz at 9.4 T to ~20 Hz at 20.0 T. This is of a similar magnitude to the J coupling (predicted by DFT calculations to be ~30 Hz), leading to the complex multiplet patterns seen. Although some variation with field is observed (confirming the cross term is significant), the resolution is not sufficient to fit the lineshape or experimentally determine the couplings involved.

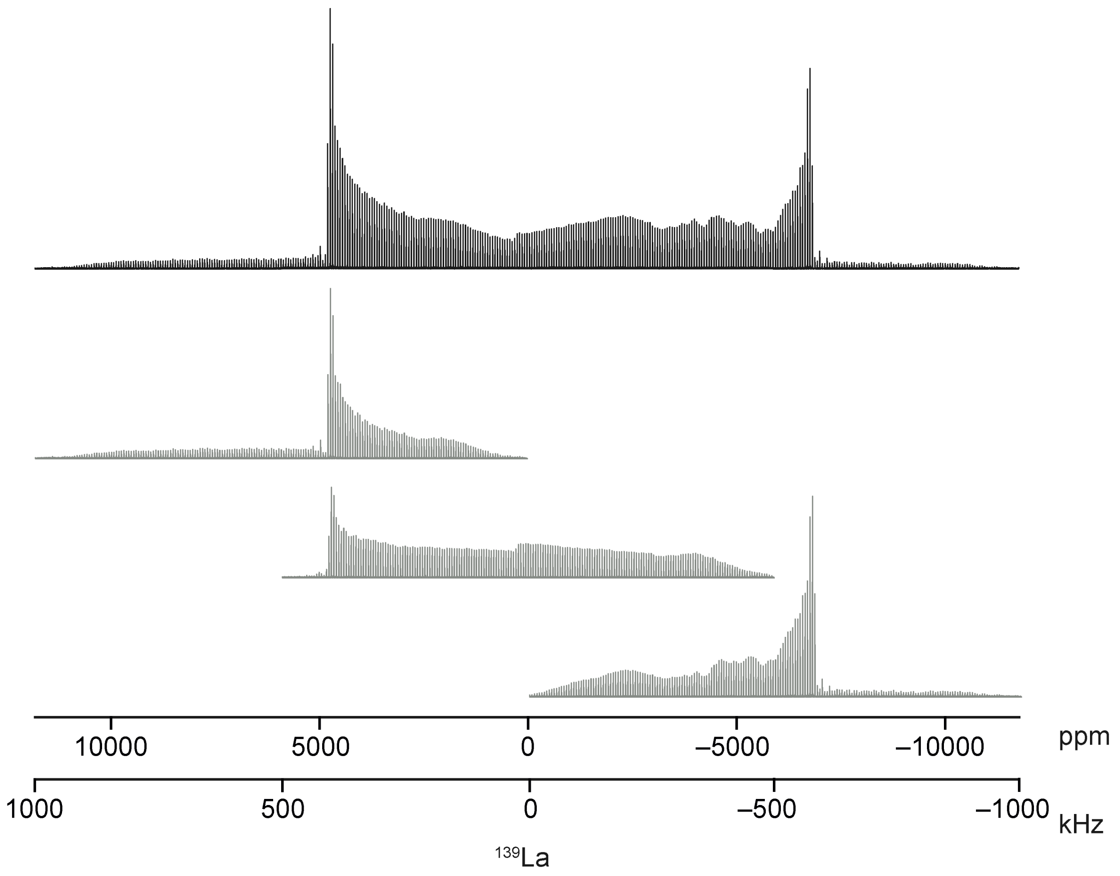

Figure S5.2 ^{139}La (14.1 T) frequency-stepped CPMG spectrum of a static sample of $La_2Sn_2O_7$. The spectrum is the result of averaging 128 transients (each containing 200 echoes) with a recycle interval of 5 s.

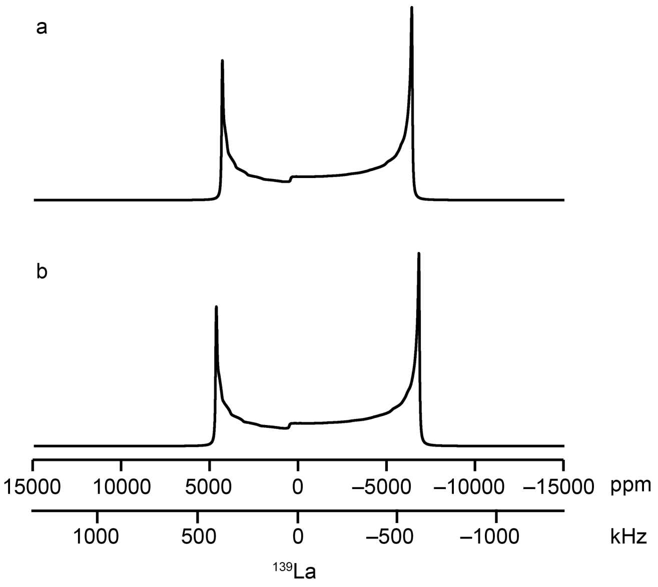

Figure S5.3 ^{139}La (14.1 T) spectrum simulated using NMR parameters predicted by DFT calculations. In (a), only the quadrupolar coupling is considered, while in (b), the CSA is also included. C_Q = 76.4 MHz, η_Q = 0, Δ_{CS} = 249 ppm, η_{CSA} = 0, (α, β, γ) = (13°, 180°, 13°).

S6. NMR of $Y_2Ti_2O_7$ and $La_2Sn_2O_7$ enriched under different conditions

Figures S6.1 and S6.2 show ^{17}O MAS NMR spectra of $Y_2Ti_2O_7$ and $La_2Sn_2O_7$, respectively, enriched (a) for 12 h at temperatures between 500 °C and 950 °C and (b) at 600 °C for the durations shown. The relative (integrated) intensity ratios, O1:O2, are shown on the figures and plotted in Figure 4 of the main text.

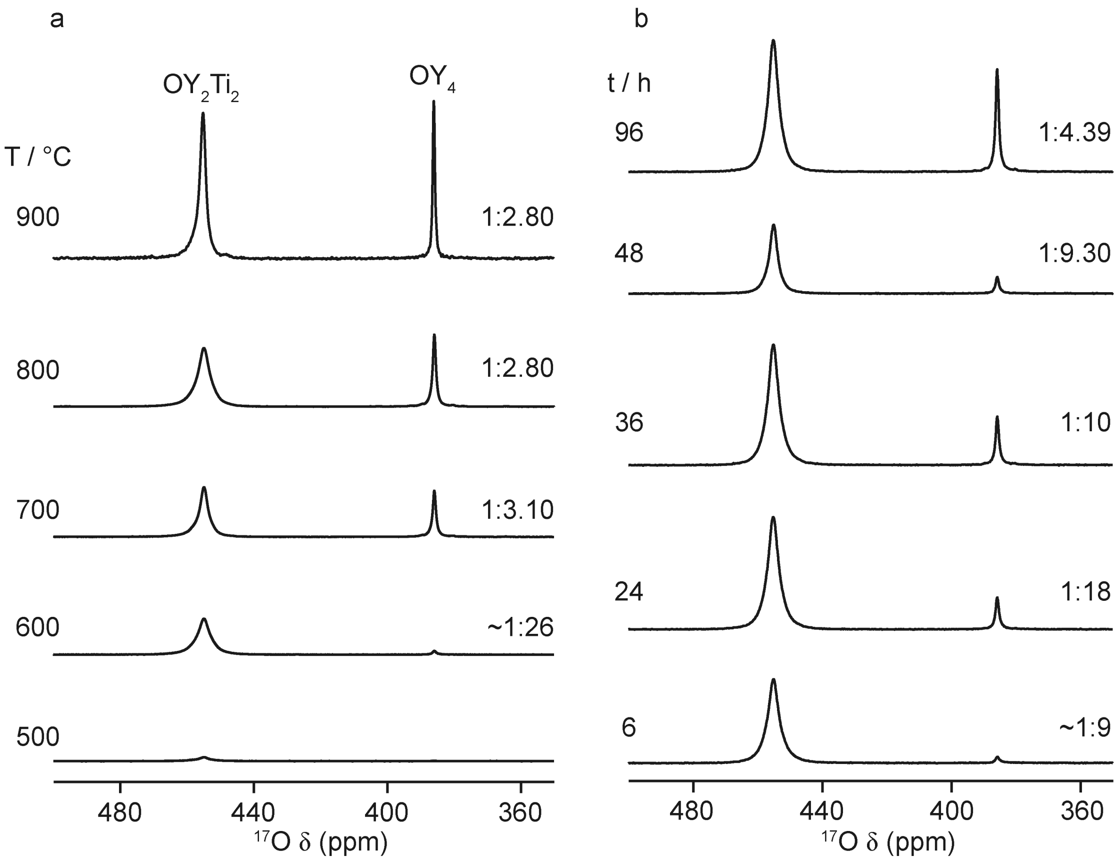

Figure S6.1 ^{17}O (14 T, 21 kHz) MAS NMR spectra of $Y_2Ti_2O_7$ enriched (a) for 12 h at the temperatures shown and (b) at 600 °C for the durations shown. Spectra were acquired using the same mass of sample and by averaging the same number of transients

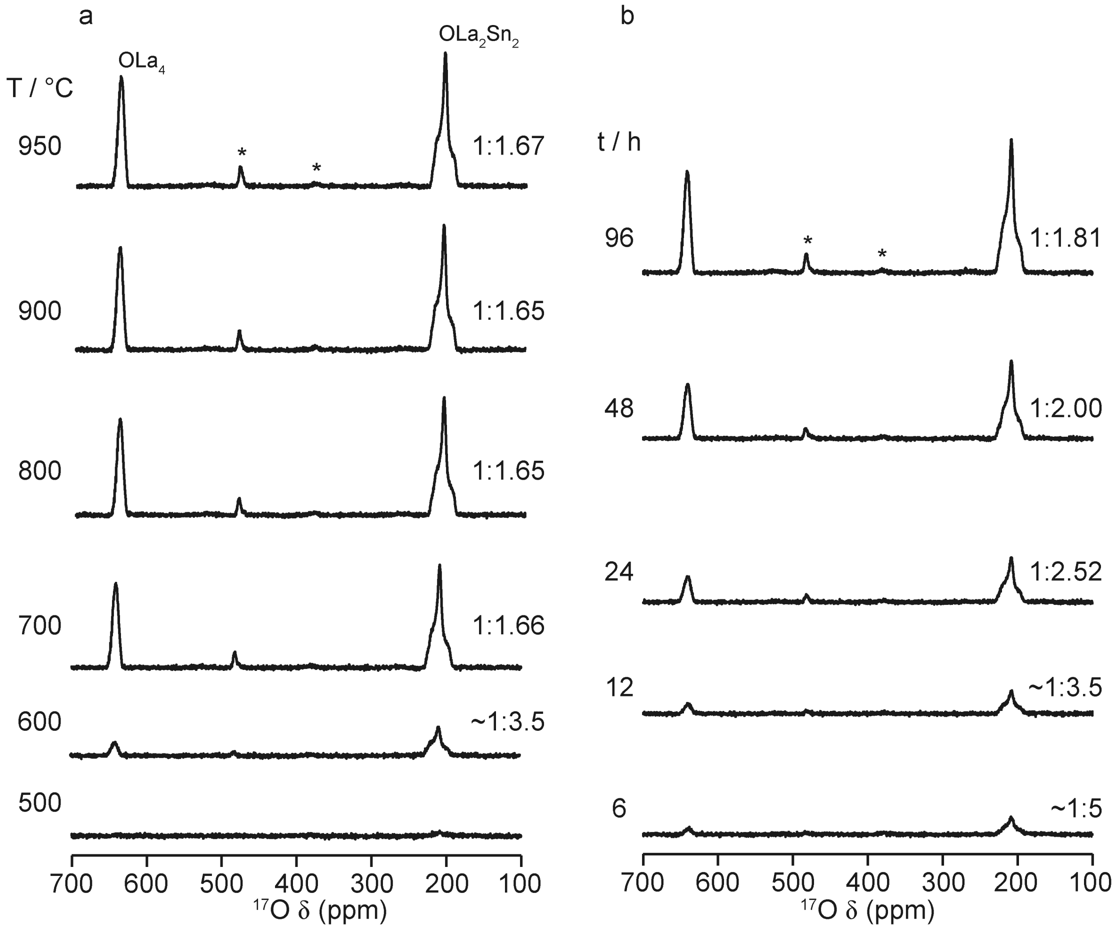

Figure S6.2 ^{17}O (14 T, 21 kHz) MAS NMR spectra of $La_2Sn_2O_7$ enriched (a) for 12 h at the temperatures shown and (b) at 600 °C for the durations shown. Spinning sidebands are marked *. Spectra were acquired using the same mass of sample and by averaging the same number of transients

S7. Nutation simulations and correction to spectral intensities

Figure S7.1 shows nutation profiles for a ^{17}O conventional single pulse NMR experiment simulated using SIMPSON[S45] with B_0 = 14.1 T, an MAS rate of 20 kHz and rf field strength of 71 kHz and C_Q values similar to those determined from experiment (Table 1 of the main text). Although differences in nutation behaviour are observed at longer pulse durations, for short pulses the excitation is quantitative. As a pulse duration of 0.5 μs was used in all experiments, no correction for nutation effects was required.

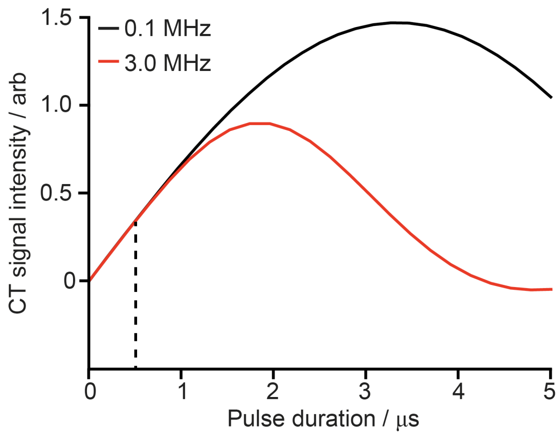

Figure S7.1 CT signal intensity (arbitrary units) in ^{17}O NMR experiments simulated with B_0 = 14.1 T, an MAS rate of 20 kHz and rf field strength of 71 kHz, for C_Q values of 0.1 (black) and 3.0 (red) MHz.

The contribution of the ST to the signals seen experimentally was determined using the density matrix simulation program SIMPSON. Spectra were simulated at 14.1 T at an MAS rate of 20 or 21 kHz, using a pulse duration of 0.5 μs (rf field strength of 71 kHz) and detection operators of I_{1x} (*i.e.*, all ^{17}O single-quantum coherences from the central transition (CT) and satellite transitions (ST)) or I_{1c} (*i.e.*, only ^{17}O CT coherences).

S8. Overall levels of ^{17}O enrichment

It is difficult to accurately compare the absolute levels of enrichment achieved for samples enriched under different conditions, owing primarily to the variation in the amount of gas used in each enrichment experiment, and also to differences in the masses of the sample studied and in the microstructure and grain size of (i) different pyrochlores and (ii) different batches (*i.e.*, synthesised at different times) of the same pyrochlore. (Note SEM images are shown in Section S1). While it is not easy to estimate the experimental uncertainty this produces in any one measurement, it is possible to consider the overall trends in absolute enrichment level (with varying time and temperature). Figure S8.1 shows the variation in absolute signal intensity (for O1 and O2 species) in the ^{17}O MAS NMR spectra of $Y_2Sn_2O_7$, $Y_2Ti_2O_7$ and $La_2Sn_2O_7$ shown in Figure 3a of the main text and Figures S6.1a and S6.2a (corrected as far as possible for differences in sample mass and normalised to the intensity obtained at the highest temperature for each material). For $Y_2Sn_2O_7$ and $La_2Sn_2O_7$, the enrichment level rises sharply after ~600 °C, reaching a maximum value at temperatures above 850 °C. In contrast, for $Y_2Ti_2O_7$, the enrichment level continues to rise with increasing temperature (and may not have reached its maximum at the highest temperature available).

Figure S8.2 shows a similar plot of the signal intensity for the ^{17}O spectra of $Y_2Sn_2O_7$, $Y_2Ti_2O_7$ and $La_2Sn_2O_7$ shown in Figure 3b of the main text and Figures S6.1b and S6.2b, as a function of heating duration, normalised to the intensity obtained at the longest heating durations for each material. For $La_2Sn_2O_7$, the absolute signal intensity increases as the heating duration increases (and may well have not reached a maximum at the longest duration used). For $Y_2Ti_2O_7$, the intensity of the O1 signal increases rapidly up to ~24 h and more slowly after this point (note the values obtained for a 48 h are anomalously low due to a smaller volume of gas being used in the experiment). For $Y_2Sn_2O_7$, enrichment of O2 appears to be high even at low heating times, but the absolute level of enrichment for O1 increases significantly with the heating duration (up to times of ~48 h). The values

obtained for 96 h heating also appear to be lower than expected, again owing to a problem with the amount of gas used in the enrichment process.

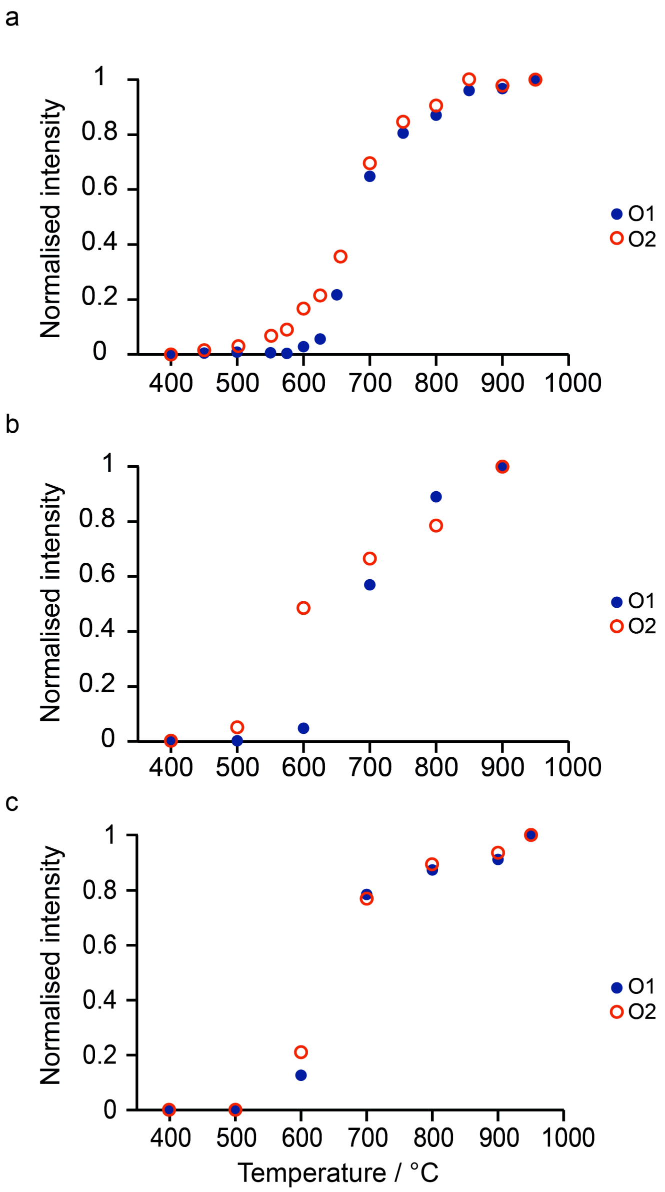

Figure S8.1 Plot of signal intensity (for O1 and O2 species) in the ^{17}O MAS NMR spectra of (a) $Y_2Sn_2O_7$, (b) $Y_2Ti_2O_7$ and (c) $La_2Sn_2O_7$ shown in Figure 3a of the main text and Figures S6.1a and S6.2a. Data have been normalised to the intensity obtained at the highest temperature for each material.

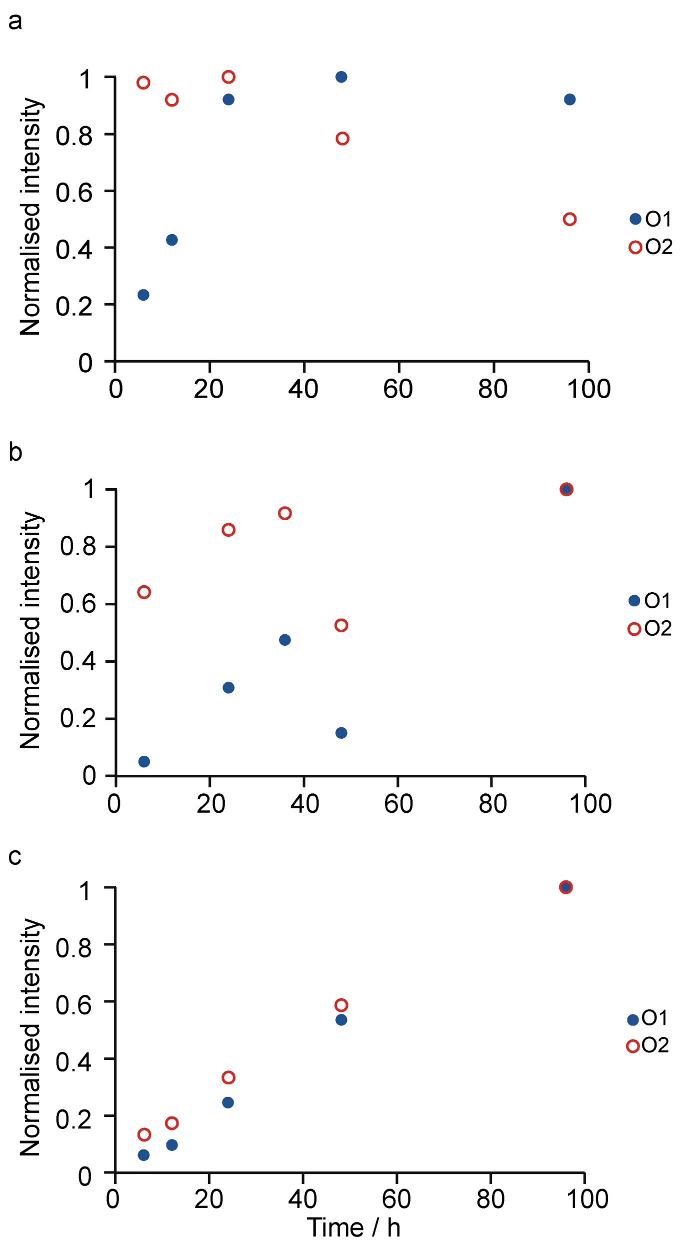

Figure S8.2 Plot of signal intensity (for O1 and O2 species) in the ^{17}O MAS NMR spectra of $Y_2Sn_2O_7$, $Y_2Ti_2O_7$ and $La_2Sn_2O_7$ shown in Figure 3b of the main text and Figures S6.1b and S6.2b. Data have been normalised to the intensity obtained at the highest temperature.

S9. Additional ^{17}O NMR spectra of $Y_2Hf_2O_7$ and impurity phases

Figure S9.1 shows ^{17}O MAS NMR spectra of $Y_2Hf_2O_7$ enriched at 600 °C for different durations. There is very little change observed in the spectral lineshape.

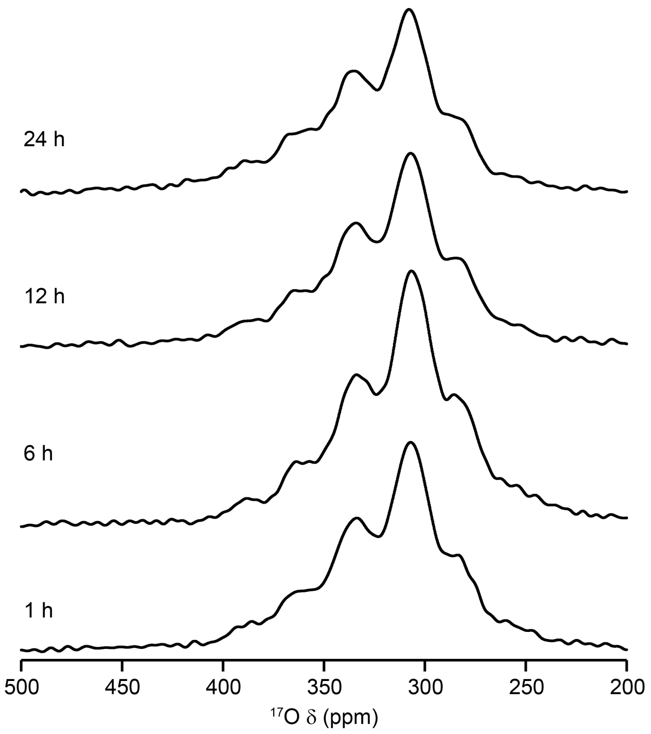

Figure S9.1 ^{17}O (9.4 T, 14 kHz MAS) NMR spectra of $Y_2Hf_2{}^{17}O_7$ enriched at 600 °C between 1 and 24 h.

Figure S9.2 shows ^{17}O MAS NMR spectra of $Y_2Hf_2O_7$ (enriched at 950 °C for 48 h) acquired at different B_0 field strengths. Although very small changes in position are observed (reflecting the increase in the (small) quadrupolar shifts at lower field), very little difference is observed in the lineshape. When considered alongside the ^{17}O MQMAS spectrum (shown in the main text) this confirms that the six distinct features seen in the ^{17}O spectrum of $Y_2Hf_2O_7$ do not result from the presence of quadrupolar powder-pattern lineshapes.

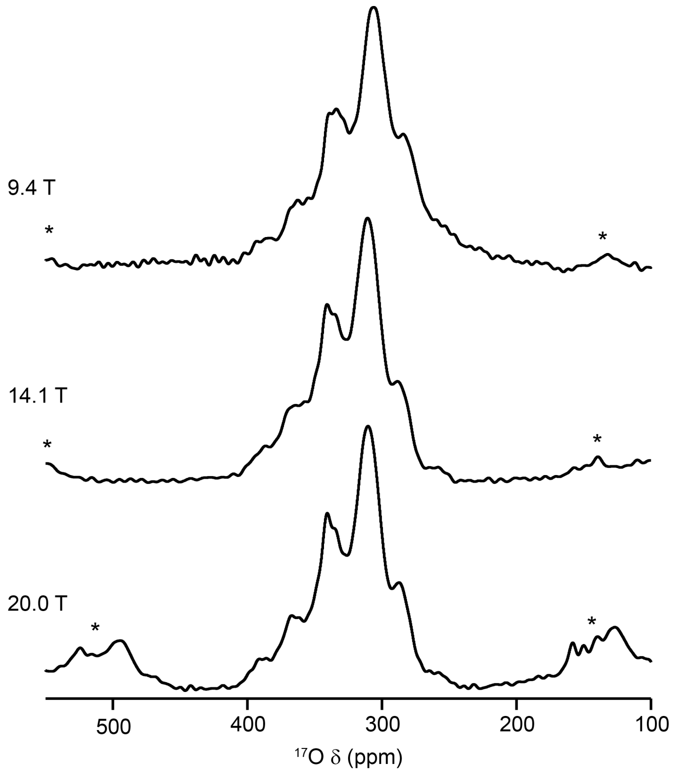

Figure S9.2 ^{17}O MAS NMR spectra of $Y_2Hf_2O_7$ (enriched at 950 °C for 48 h) at magnetic field strengths of 9.4 (14.0 kHz), 14.1 (21.0 kHz) and 20.0 T (21.0 kHz). * denote spinning sidebands.

Figure S9.3 compares the ^{17}O (14.1 T) MAS NMR spectrum of $Y_2Hf_2O_7$ (enriched at 950 °C for 48 h) with spectra of possible impurity phases: monoclinic HfO_2 (enriched at 600 °C for 12 h), Y_2O_3 (enriched at 600 °C for 12 h), and the background from the ZrO_2 rotor (natural abundance). There is some evidence for a small (~1%) HfO_2 impurity in the sample, and some suggestion that an even smaller Y_2O_3 impurity could be present. The signal expected from the rotor (ZrO_2) does overlap with the spectral lineshape of $Y_2Hf_2O_7$, but experiments on a rotor containing no ^{17}O-enriched material confirmed this (natural abundance) background signal does not contribute to the spectra at the experimental conditions and times used.

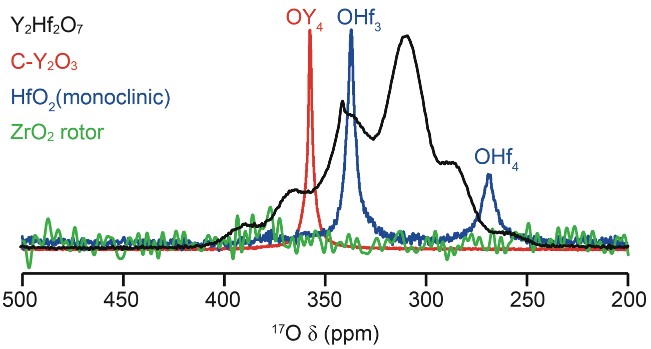

Figure S9.3 ^{17}O (14.1 T, 21 kHz) MAS NMR spectra of $Y_2Hf_2O_7$ (black), HfO_2 (blue), cubic Y_2O_3 (red) and the background from the ZrO_2 rotor (green).

Figure S9.4a compares ^{17}O MAS NMR spectra of $Y_2Hf_2O_7$ acquired with different recycle intervals. Little difference is seen in the relative intensities of the spectral lines, demonstrating (i) that they are an accurate reflection of the relative amounts of ^{17}O in each environment and (ii) that the signals do not result from a paramagnetic impurity (which may also cause a change in resonance position).

Signal intensity resulting from the central transition (CT) can be distinguished from that resulting from any satellite transitions (ST) by adjustment of the angle of the spinning axis away from the magic angle. This will lead to a broadening of the ST, while CT signals remain sharp. Spectra of $Y_2Hf_2O_7$ acquired with the spinning angle accurately adjusted to the magic angle, and misset from this, are compared in Figure S9.4b. It is clear from the figure that all of the six major resonances observed can be attributed to CT signal. Signal from a small impurity is also observed at lower shift (~180 ppm)

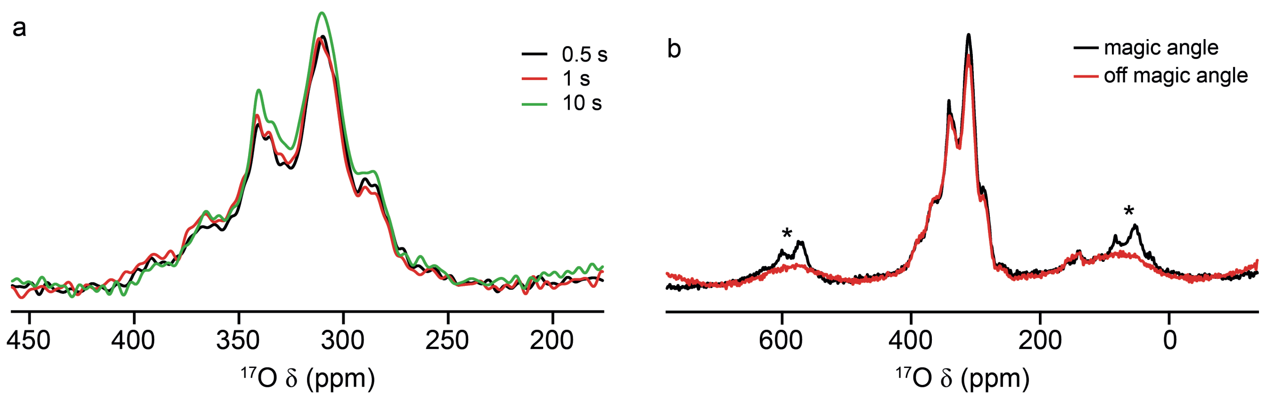

Figure S9.4 (a) ^{17}O (20.0 T, 21 kHz) MAS NMR spectra of $Y_2Hf_2O_7$, 950 °C for 24 h, acquired with different recycle intervals. (b) ^{17}O (14.1 T, 21 kHz) MAS NMR spectra of $Y_2Hf_2O_7$, 950 °C for 48 h, acquired with the spinning angle accurately set to the magic angle (black) and misset from this (red). * denote spinning sidebands.

As hydroxyl O species often have much larger C_Q values (*e.g.*, 4-8 MHz) it is unlikely that any of the signals in the ^{17}O MAS NMR spectrum of $Y_2Hf_2O_7$ result from surface –OH species (potentially observable owing to more preferential enrichment of the surface of the particles). ^{1}H-^{17}O CP experiments did not result in any signal, confirming this is unlikely to be the origin of any signal seen.

Figure S9.5 compares the same spectrum of $Y_2Hf_2O_7$ with the ^{17}O spectra of $Y_2Zr_2O_7$, $Y_2Sn_2O_7$ and $Y_2Ti_2O_7$ shown in the main text. All three of these are expected to possess an OY_4 environment, and for the two pyrochlore samples these are seen at 384 and 386 ppm, in good agreement with the resonance observed at ~387 ppm for $Y_2Hf_2O_7$. The signal for $Y_2Zr_2O_7$ covers this region of the spectrum, but is centered downfield, reflecting the increase in electronegativity of Zr (1.33) compared to Y (1.22), which reduces the ionicity of the O-Zr bond relative to the O-Y bond. As Hf possesses a similar, but slightly lower, electronegativity (1.3) the reduced ionicity of the O-Hf bonds would also be expected to result in an upfield shift of resonances and Hf neighbours were introduced, in agreement with the spectrum observed experimentally.

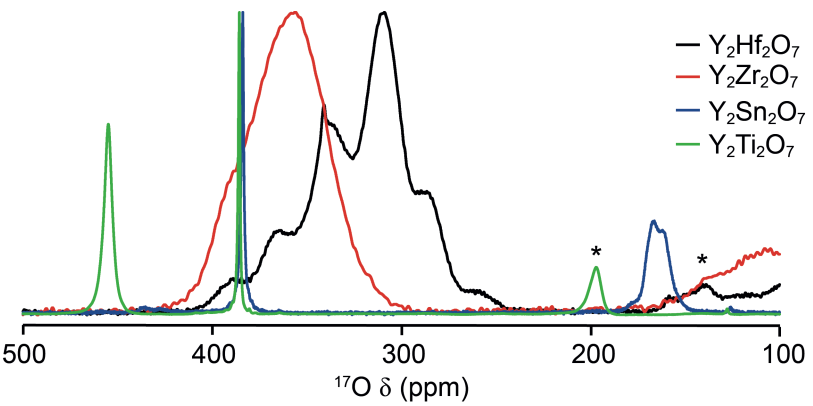

Figure S9.5 ^{17}O (14.1 T, 21 kHz) MAS NMR spectra of $Y_2Hf_2O_7$ (black), $Y_2Zr_2O_7$ (red), $Y_2Sn_2O_7$ (blue) and $Y_2Ti_2O_7$ (green). * denote spinning sidebands.

S10. DFT calculations for $Y_2Zr_2O_7$ and $Y_2Hf_2O_7$

As described in the main text, for a "unit cell" of similar size to that of a pyrochlore (*i.e.*, containing 32 cation sites (occupied by 16 Y and 16 Zr/Hf) and 64 anion sites (occupied by 56 oxygens and 8 vacancies), there ~2.64×10^{18} possible defect fluorite structures (resulting from ~600 million possible cation arrangements and ~4.4 billion possible anion arrangements). Even when restricting this to symmetry-unique environments it is clearly unfeasible to consider all of these (or even any statistically-relevant subset).

A set of 30 $Y_2Zr_2O_7$ defect fluorite structural models was produced, starting from a 2 × 2 × 2 supercell of ideal AO_2 fluorite. Using a FORTRAN script, 16 of the 32 cation sites were randomly selected to be occupied by Y, with the remainder occupied by Zr. Similarly, 8 of the 64 anion sites were randomly selected to be vacant (and the remaining 56 occupied by O). The structures were geometry optimised using DFT. (It should be noted that many of these structural models were far from equilibrium and required the geometry optimisation to be carried out in two different stages; the first using the EDFT SCF solver in CASTEP for a maximum of 50 optimisation steps, and the second taking the output from the first calculation and using the DM SCF solver for up to 400 optimisation iterations). The duration of the first optimisation step was typically ~35 h (on 224 cores), the second typically ~120 h (on 64 cores). NMR parameters were then calculated (taking typically ~7 h on 64 cores). In addition, a further 4 structural models were considered. The first was a $Y_2Zr_2O_7$ pyrochlore structure (*i.e.*, with ordering of cations and anions) generated by atom substitution from a structural model of $Y_2Sn_2O_7$. Two models were generated from this; one by moving one 8a O to a vacant 8b site (creating a seven-coordinate Y species and an OZr_4 environment), and a second by moving another 8a O from around the same Y to a vacant 8b site (creating a six-coordinate Y species). The final structural model was a reverse pyrochlore structure, *i.e.*, containing Y on the B site and Zr on the A site. All structures were geometry optimised using only the second optimisation procedure described above. For $Y_2Hf_2O_7$, 30 defect fluorite models were obtained by substituting Zr for Hf in the structures described above and subjecting these to further

geometry optimisation. The four corresponding Hf-containing pyrochlore structures were generated once more from $Y_2Sn_2O_7$.

The structural models produced span an energy range of 5.35 eV for $Y_2Zr_2O_7$ and 5.42 eV for $Y_2Hf_2O_7$, and five-, six-, seven- and eight-coordinate Y species are present. For $Y_2Zr_2O_7$, the ordered pyrochlore structure was 0.41 eV higher in enthalpy than the lowest disorder structure (note, this does not take into account any contribution from configurational entropy). The structures produced by moving one and two 8a O species were 1.94 and 5.12 eV higher in energy, respectively, and the reverse pyrochlore 22.14 eV higher than the lowest energy structural model. This confirms the preference (also seen in the ^{89}Y NMR spectra) for Y to have a higher coordination number. The lowest energy structure produced had 2 six-coordinate, 12 seven-coordinate and 2 eight-coordinate Y species. The ^{17}O chemical shifts predicted from these structural models are shown in Figure S10.1a and calculated absolute C_Q values in Figure S10.1a. All are very similar and small, suggesting quadrupolar broadening has a limited effect in the experimental spectrum. For $Y_2Hf_2O_7$, the disordered structural model with the lowest energy had 1 six-coordinate, 13 seven-coordinate and 2 eight-coordinate Y species. Interestingly, this model was higher in energy (by 1.01 eV) than the ordered pyrochlore structure. Structures produced by moving one and two 8a O species were 1.00 and 4.70 eV higher in energy, and the reverse pyrochlore 23.37 eV higher than the lowest energy structural model. This suggests a stronger preference for higher Y coordination numbers in hafnate-based materials than in the corresponding zirconate structures. The ^{17}O chemical shifts predicted from these structural models are shown in Figure S10.1b, and the calculated absolute C_Q values in Figure S10.2b.

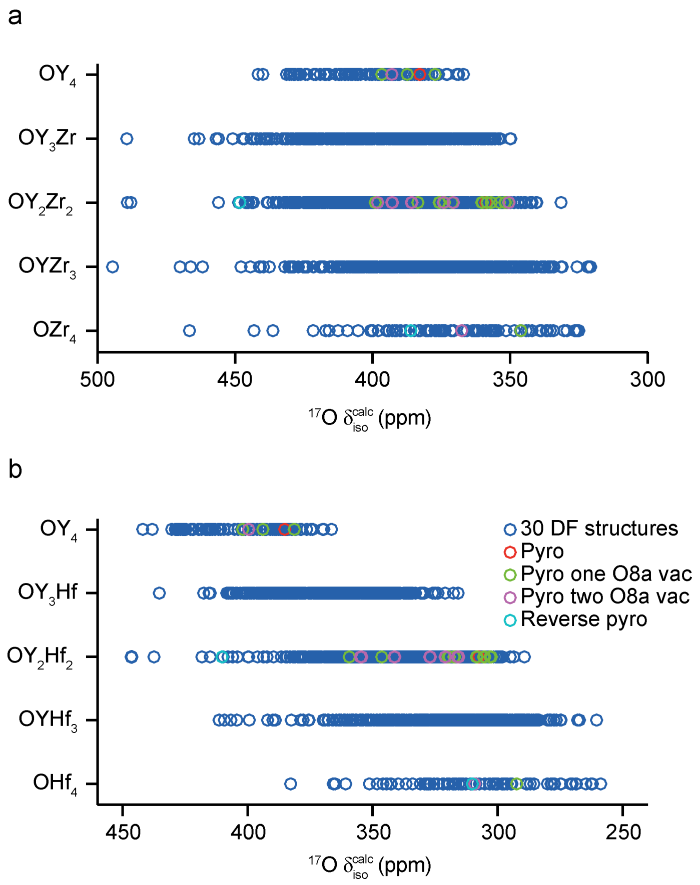

Figure S10.1 Calculated ^{17}O δ_{iso}^{calc} for ~ 30 structural models of (a) $Y_2Zr_2O_7$ and (b) $Y_2Hf_2O_7$, separated by the local atomic environment. Points corresponding to the four additional pyrochlore-based structural models (described above) are highlighted.

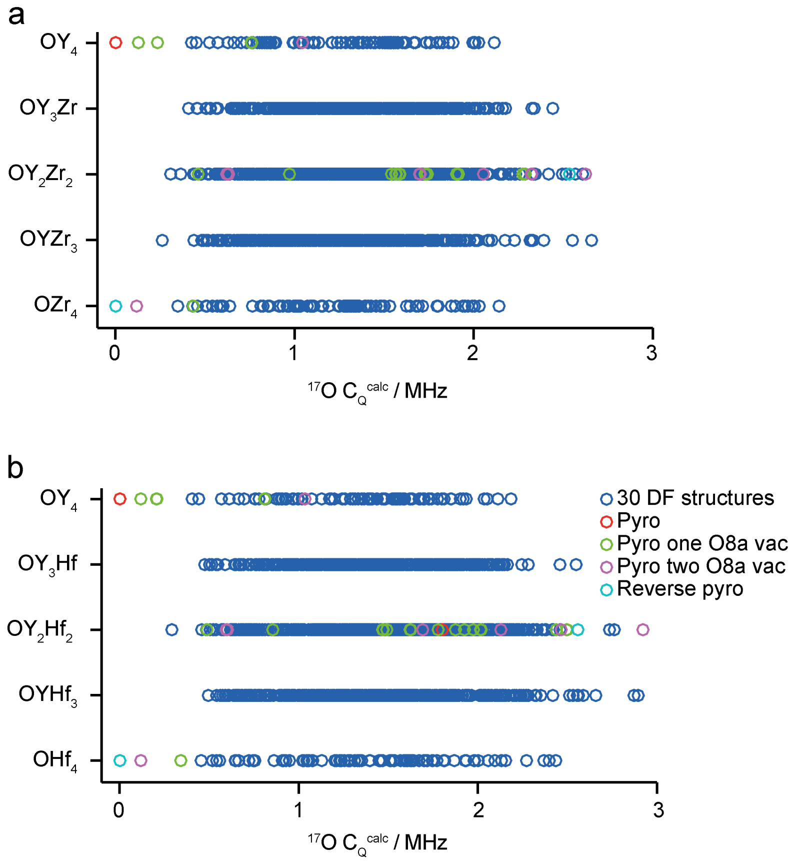

Figure S10.2 Calculated ^{17}O C_Q for ~30 structural models of (a) $Y_2Zr_2O_7$ and (b) $Y_2Hf_2O_7$, separated by the local atomic environment. Points corresponding to the four additional pyrochlore-based structural models (described above) are highlighted.

S11. ^{17}O NMR of $La_2Ti_2O_7$

Figure S11.1 shows the variation in absolute signal intensity in the ^{17}O MAS NMR spectra of $La_2Ti_2O_7$ shown in Figure 9a of the main text (corrected as far as possible for differences in sample mass, and normalised to the intensity obtained at the highest temperature).

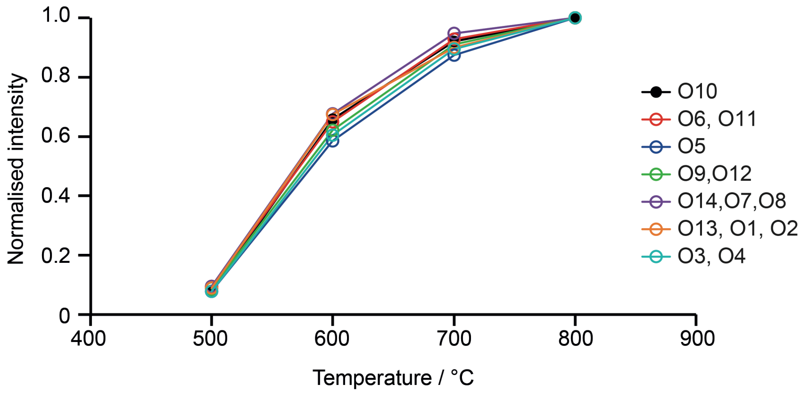

Figure S11.1 Plot of signal intensity in the ^{17}O (14.1 T, 20 kHz) MAS NMR spectra (integrated over all O species) of $La_2Ti_2O_7$ shown in Figure 9a of the main text. Data have been normalised to the intensity obtained at the highest temperature.

Figure S11.2 shows ^{17}O MAS (14.1 T, 21 kHz) of $La_2Ti_2O_7$ enriched at 600 °C for varying durations. Little difference is seen in the relative intensities of the spectral resonances.

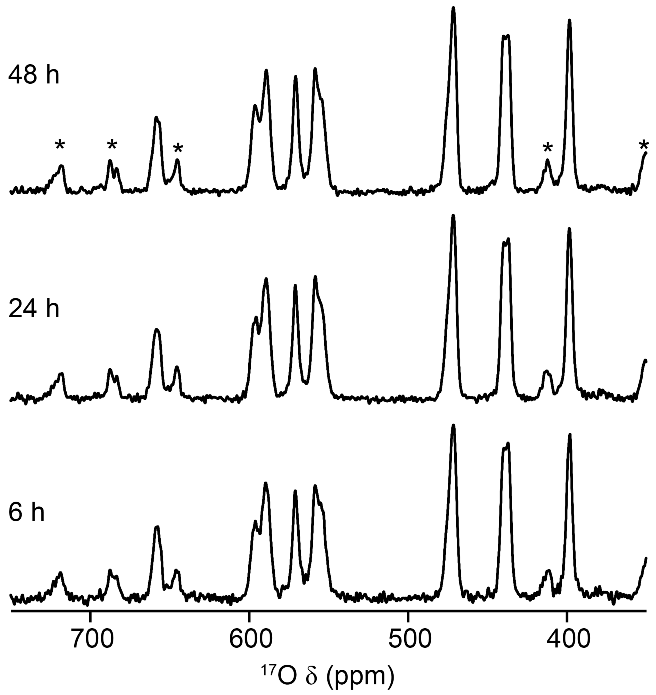

Figure S11.2 ^{17}O (14.1 T, 21 kHz) MAS NMR spectra of $La_2Ti_2O_7$ enriched at 600 °C for varying durations. Data have been normalised to the intensity obtained at the highest temperature. * denotes spinning sidebands.

S12. DFT calculations for $La_2Ti_2O_7$

DFT calculations were carried out for the monoclinic and orthorhombic structural models of $La_2Ti_2O_7$. When the referencing (and scaling) procedure outlined in Section S3 was applied, poor agreement with experiment was observed. Figure S12.1 shows the plot of calculated isotropic shieldings (σ_{iso}^{calc}) and experimental isotropic shifts (δ_{iso}^{exp}) shown previously in Figure S3.1a, but with the 14 points for (monoclinic) $La_2Ti_2O_7$ also added (in red). Here, the experimental and calculated shifts have simply been ordered and plotted against each other (see main text for discussion). While the new points are in reasonably good agreement with the previous data, particularly given the larger scatter at high δ_{iso}^{exp}, it is clear from a linear regression, that when only the red data points are considered, these lie on a line with a different gradient (and hence a different scaling factor), which would lead to a very different reference when considered in isolation. This might result from issues with the pseudopotentials used for La and Ti (likely to be less well tested for NMR calculations than more commonly studied species, or perhaps from the lack of a full consideration of relativity. Given the uncertainty over the structural model for $La_2Ti_2O_7$[S45-S46] it does not seem sensible to include these points in any determination of reference value and/or and scaling factor, but to facilitate comparison with experiment (and interpretation of the experimental spectra), DFT calculations were referenced using only the peaks seen in the experimental spectrum. The reference value and scaling factor were determined using the peaks at highest and lowest shift only (thereby removing any uncertainty over spectral assignment in the more crowded regions of the spectrum).

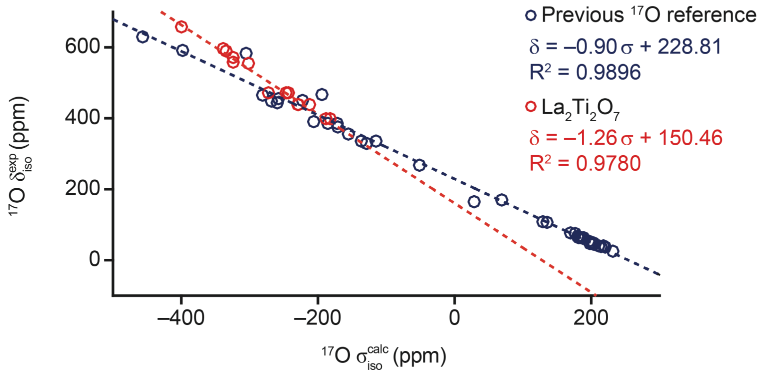

Figure S12.1 Plot of δ_{iso}^{exp} against σ_{iso}^{calc} for shown previously in Figure S3.1a, with points for (monoclinic) $La_2Ti_2O_7$ added in red.

Table S12.1 shows the calculated NMR parameters for the monoclinic and orthorhombic structural models of $La_2Ti_2O_7$, obtained after optimisation of each model. When determining the type of O environment all cations with 3, 3.5 and 4 Å are given. Figure S12.2 shows the position of each type of O species within the structure of the two structural models.

Table S12.1 Calculated ^{17}O NMR parameters for monoclinic and orthorhombic structural models of $La_2Ti_2O_7$.

Species[a]	Species[b]	Environment 3 Å	Environment 3.5 Å	Environment 4.0 Å	δ_{iso}^{calc} (ppm)	C_Q^{calc} / MHz
Monoclinic model of $La_2Ti_2O_7$[S46]						
O1		OLa_2Ti_2	OLa_3Ti_2	OLa_3Ti_2	453.0	1.06
O2		OLa_2Ti_2	OLa_3Ti_2	OLa_4Ti_2	433.8	1.74
O3		OLa_3Ti_2	OLa_4Ti_2	OLa_4Ti_2	404.8	0.94
O4		OLa_3Ti_2	OLa_4Ti_2	OLa_4Ti_2	398.0	0.98
O5		OLa_2Ti_2	OLa_3Ti_2	OLa_4Ti_2	567.2	0.44
O6		OLa_2Ti_2	OLa_4Ti_2	OLa_4Ti_2	584.4	0.27
O7		OLa_2Ti_2	OLa_2Ti_2	OLa_3Ti_2	470.8	1.23
O8		OLa_2Ti_2	OLa_2Ti_2	OLa_3Ti_2	474.6	1.34

O9		OLa_3Ti	OLa_3Ti	OLa_3Ti	567.0	0.38
O10		OLa_2Ti	OLa_3Ti	OLa_3Ti	657.7	0.45
O11		OLa_3Ti	OLa_3Ti	OLa_3Ti	580.2	0.39
O12		OLa_3Ti	OLa_3Ti	OLa_3Ti	540.3	0.59
O13		OLa_2Ti_2	OLa_3Ti_2	OLa_3Ti_2	454.0	1.51
O14		OLa_2Ti_2	OLa_2Ti_2	OLa_3Ti_2	505.5	1.33
Orthorhombic model of $La_2Ti_2O_7$[S47]						
O9	O1	OLa_2Ti_2	OLa_3Ti_2	OLa_3Ti_2	452.5	1.06
O6	O2	OLa_2Ti_2	OLa_3Ti_2	OLa_4Ti_2	434.4	1.71
O7	O3	OLa_3Ti_2	OLa_4Ti_2	OLa_4Ti_2	412.1	0.94
O8	O4	OLa_3Ti_2	OLa_4Ti_2	OLa_4Ti_2	400.0	0.97
O4	O5	OLa_2Ti_2	OLa_3Ti_2	OLa_4Ti_2	571.6	0.40
O11	O6	OLa_2Ti_2	OLa_3Ti_2	OLa_4Ti_2	582.4	0.29
O5	O7	OLa_2Ti_2	OLa_2Ti_2	OLa_3Ti_2	469.2	1.28
O10	O8	OLa_2Ti_2	OLa_2Ti_2	OLa_3Ti_2	475.9	1.35
O2	O9	OLa_3Ti	OLa_3Ti	OLa_3Ti_2	569.6	0.36
O13	O10	OLa2Ti	OLa_3Ti	OLa_3Ti_2	657.9	0.45
O1	O11	OLa_3Ti	OLa_3Ti	OLa_3Ti	578.3	0.36
O14	O12	OLa_3Ti	OLa_3Ti	OLa_3Ti_2	541.8	0.63
O3	O13	OLa_2Ti_2	OLa_3Ti_2	OLa_3Ti_2	452.2	1.63
O12	O14	OLa_2Ti_2	OLa_2Ti_2	OLa_3Ti_2	500.4	1.38

[a] Crystallographic label from original cif file

[b] Renumbered crystallographic label to enable comparison to monoclinic structure

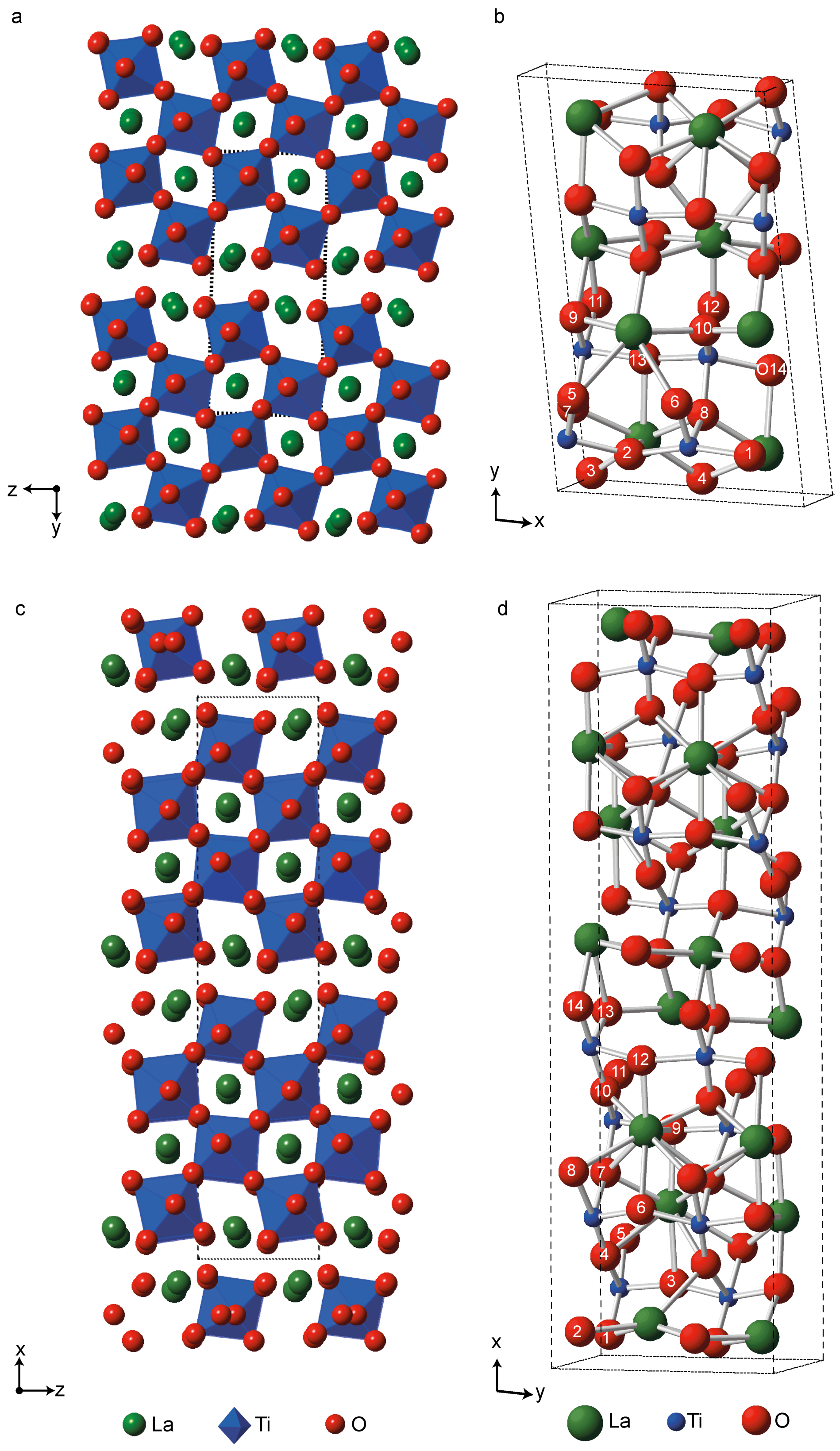

Figure S12.2 Structural models for (a, b) monoclinic and (c, d) orthorhombic $La_2Ti_2O_7$, with the crystallographic labels (in the original cif files) for each O species indicated in (b, d).

[Figure S12.3](#) plots calculated NMR parameters (δ_{iso}^{calc} and C_Q^{calc}) for the monoclinic and orthorhombic structural models of $La_2Ti_2O_7$, along with the ^{17}O MAS NMR spectrum of $La_2Ti_2O_7$ enriched at 900 °C for 12 h. There is very little difference between the calculated parameters for the two structures, and both exhibit similar agreement with experiment, suggesting that ^{17}O NMR is not able to distinguish the two unambiguously. The energy (after optimisation) of the monoclinic form was very slightly lower (by 0.44 meV) than that of the orthorhombic form. This is in agreement with the conclusion reached by Fernandes *et al.*[S48] in previous work studying Sn-substituted $La_2Ti_2O_7$.

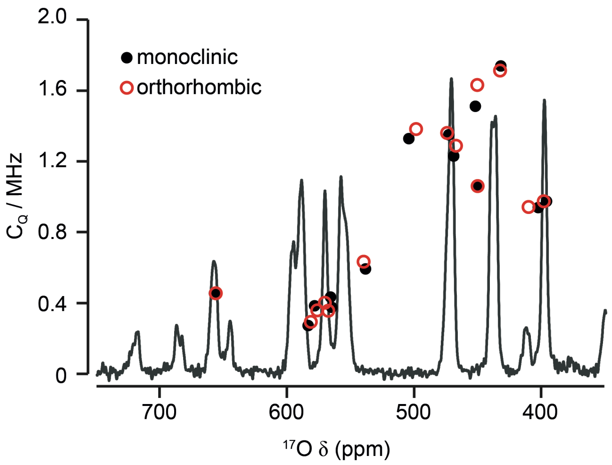

Figure S12.3 Calculated NMR parameters (δ_{iso}^{calc} and C_Q^{calc}) for the monoclinic (black filled circles) and orthorhombic (red open circles) structural models of $La_2Ti_2O_7$. Also shown is the ^{17}O MAS NMR spectrum of $La_2Ti_2O_7$ enriched at 900 °C for 12 h.

S13. References

S1. M. Profeta, M. Benoit, F. Mauri and C. J. Pickard, *J. Am. Chem. Soc*. 2004, **126**, 12628-12635.

S2. R. T. Downs and D. C. Palmer, *Am. Mineral.*, 1994, **79**, 9-14.

S3. M. Profeta, F. Mauri and C. J. Pickard, *J. Am. Chem. Soc.*, 2003, **125**, 541-548.

S4. R. H. Hazen, L. W. Finger, R. J. Hemley and H. K. Mao, *Solid State Communications*, 1989, **72**, 507-511.

S5. N. L. Ross and R. M. Hazen, *Phys. Chem. Miner.*, 1990, **17**, 228-237.

S6. S. E. Ashbrook, A. J. Berry, D. J. Frost, A. Gregorovic, C. J. Pickard, J. E. Readman and S. Wimperis, *J. Am. Chem. Soc.*, 2007, **129**, 13213-13224.

S7. H. Horiuchi, M. Hirano, E. Ito and Y. Matsui, *Am. Mineral.*, 1982, **67**, 788-793.

S8. J. R. Smyth and R. m. Hazen, *Am. Mineral.*, 1973, **58**, 588-593.

S9. S. E. Ashbrook, A. J. Berry and S. Wimperis, *Am. Mineral*., 1999, **84**, 1191-1194.

S10. S. E. Ashbrook, A. J. Berry and S. Wimperis, *J. Am. Chem. Soc.*, 2001, **123**, 6360-6366.

S11. S. E. Ashbrook, A. J. Berry, W. O. Hibberson, S. Steuernagel and S. Wimperis, *Am. Mineral.*, 2005, **90**, 1861-1870.

S12. H. Horiuchi and H. Sawamoto, *Am. Mineral.*, 1981, **66**, 568-575.

S13. S. E. Ashbrook, A. J. Berry, W. O. Hibberson, S. Steuernagel and S. Wimperis, *J. Am. Chem. Soc.*, 2003, **125**, 11824-11825.

S14. S. Sasaki, C. T. Prewitt, Y. Sato and E. Ito, *J. Geophys. Res.*, 1982, **87**, 7829-7832.

S15. Yu. A. Abramov, V. G. Tsirel'son, V. E. Zavodnik, S. A. Ivanov, S.A and L. D. Brown, *Acta Crystallogr. B*, 1995, **51**, 942-951.

S16. T. J. Bastow, P. J. Dirken, M. E. Smith and H. J. Whitfield, *J. Phys. Chem.*, 1996, **100**, 18539-18545.

S17. T. Pagnier, I. Charrier-Cougoulic, C. Ritter and G. Lucazeau, *Eur. Phys. J. AP*, 2000, **9**, 1-9.

S18. S. C. Abrahams, J. M. Reddy and J. L. Bernstein, *J. Phys. Chem. Solids*, 1966, **27**, 997-1012.

S19. H. Lehnert, H. Boysen, J. Schneider, F. Frey, D. Hohlwein, P. Radaelli and H. Ehrenberg, *Z. Kristallogr*, 2000, **215**, 536-541.

S20. F. Brisse and O. Knop, *Can. J. Chem.*, 1968, **46**, 859-873.

S21. N. Kim and C. P. Grey, *J. Solid State Chem.*, 2003, **175**, 110-115.

S22. O. Knop, F. Brisse and L. Castelliz, *Can. J. Chem.*, 1969, **47**, 971-990.

S23. M. G. Patson and E. N. Maslen, *Acta. Crystallogr.*, 1965, **19**, 307-310.

S24. E. Oldfield, C. Coretsopoulos, S. Yang, L. Reven, H. C. Lee, J. Shore, O. H. Han and E. Ranli, *Phys. Rev. B*, 1989, **40**, 6832-6849.

S25. B. H. Baur and A. A. Khan, *Acta Crystallogr.* B, 1971, **27**, 2133-2139.

S26. T. J. Bestow, A. F. Moodie, M. E. Smith and H. J. Whitfield, *J. Mater. Chem.*, 1993, **3**, 697-702.

S27. H. J. A. Koopmanns, G. M. H. van de Velde and P. J. Gellings, *Acta Crystallogr. C,* 1983, **39**, 1323-1325.

S28. J. W. Downs, F. K. Ross and G. V. Gibbs, *Acta Crystallogr. B,* 1985, **41**, 425-431.

S29. G. L. Turner, S. E. Chung and E. Oldfield, *J. Magn. Reson*, 1985, **64**, 316-324.

S30. V. G. Tsirelson, A. S. Avilov, Y. A. Abramov, E. L. Belokoneva, R. Kitaneh and D. Feil, *Acta Crystallogr. B*, 1998, **54**, 8-17.

S31. M. C. Verbraeken, E. Suard and J. T. S. Irvine, *J. Solid State Chem.*, 2011, **184**, 2088-2096.

S32. M. C. Verbraeken, E. Suard and J. T. S. Irvine, *J. Mater. Chem.*, 2009, **19**, 2766-2770.

S33. W. Gerlach, *Zeitschrift fuer Physik*, 1922, **9**, 184-192.

S34. S. C. Abrahams and J. L. Bernstein, *Acta Crystallogr. B*, 1969, **25**, 1233-1236.

S35. G. Malmros, *Acta Chem. Scand.*, 1970, **24**, 384-396.

S36. K. Aurivillius, *Acta Chem. Scand.*, 1964, **18**, 1305-1306.

S37. S. C. Abrahams and J. Kalnajs, *Acta Crystallographica*, 1954, **7**, 838-842.

S38. L. Marsella and V. Florentini, *Phys. Rev. B: Condens. Matter*, 2004, **69**, 172103-172104.

S39. R. Ruh and P. W. R. Corfield, *J. Am. Ceram. Soc.*, 1970, **53**, 126-129.

S40. T. J. Bastow, M. E. Smith and H. J. Whitfield, *J. Mater. Chem.*, 1996, **6**, 1951-1955.

S41. Y. Ohashi, *Phys. Chem. Minerals*, 1984, **10**, 217-229.

S42. S. E. Ashbrook, A. J. Berry and S. Wimperis, *J. Phys. Chem. B*, 2002, **106**, 773-778.

S43. R. K. Harris and A. C. Olivieri, *Prog. NMR Spectrosc.*, 1992, **24**, 435-456.

S44. S. E. Ashbrook, J. McManus, M. J. Thrippleton and S. Wimperis, *Prog. Nucl. Magn. Reson. Spectrosc.*, 2009, **55**, 160-181.

S45. M. Bak, J. Rasmussen, N. Nielsen, *J. Magn. Reson.*, 2000, **147**, 296-330.

S46. P. R. Gasperin, *Acta Crystallogr., Sect. B: Struct. Crystallogr. Cryst. Chem.* 1975, **31**, 2129-2130.

S47. K. Scheunemann and H. B. Mueller-Buschbaum, *J. Inorg. Nucl. Chem.* 1975, **37**, 1879-1881.

S48. A. Fernandes, D. McKay, S. Sneddon, D. M. Dawson, S. Lawson, R. Veazey, K. R. Whittle and S. E. Ashbrook, *J. Phys. Chem. C*, 2016, **120**, 20288-20296.
